# Preventive effects of folic acid on Zika virus-associated poor pregnancy outcomes in immunocompromised mice

**DOI:** 10.1371/journal.ppat.1008521

**Published:** 2020-05-11

**Authors:** Yogy Simanjuntak, Hui-Ying Ko, Yi-Ling Lee, Guann-Yi Yu, Yi-Ling Lin

**Affiliations:** 1 Institute of Biomedical Sciences, Academia Sinica, Taipei, Taiwan; 2 Graduate Institute of Microbiology and Public Health, College of Veterinary Medicine, National Chung-Hsing University, Taichung, Taiwan; 3 National Institute of Infectious Diseases and Vaccinology, National Health Research Institutes, Zhunan, Taiwan; National Institute of Allergy and Infectious Diseases, UNITED STATES

## Abstract

Zika virus (ZIKV) infection may lead to congenital microcephaly and pregnancy loss in pregnant women. In the context of pregnancy, folic acid (FA) supplementation may reduce the risk of abnormal pregnancy outcomes. Intriguingly, FA may have a beneficial effect on the adverse pregnancy outcomes associated with ZIKV infection. Here, we show that FA inhibits ZIKV replication in human umbilical vein endothelial cells (HUVECs) and a cell culture model of blood-placental barrier (BPB). The inhibitory effect of FA against ZIKV infection is associated with FRα-AMPK signaling. Furthermore, treatment with FA reduces pathological features in the placenta, number of fetal resorptions, and stillbirths in two mouse models of *in utero* ZIKV transmission. Mice with FA treatment showed lower viral burden and better prognostic profiles in the placenta including reduced inflammatory response, and enhanced integrity of BPB. Overall, our findings suggest the preventive role of FA supplementation in ZIKV-associated abnormal pregnancy and warrant nutritional surveillance to evaluate maternal FA status in areas with active ZIKV transmission.

## Author summary

Zika virus (ZIKV) infection in pregnant women may cause a broad spectrum of abnormal pregnancy outcomes. Folic acid (FA) is an essential water-soluble vitamin B required for placental and fetal development to avoid placental abruption, neural tube defects, intrauterine growth restriction, and pregnancy loss. However, the beneficial role of FA in improving clinical presentations with *in utero* transmission of ZIKV is unknown. We observe that FA treatment increases the level of maternal serum FA and improves feto-placental outcomes in two mouse models of *in utero* transmission of ZIKV. FA blocks ZIKV transmission in a cell culture model of blood-placental barrier by inhibiting viral replication in endothelial cells. This study may serve as a scientific reference for public health practitioners to evaluate the FA status of pregnant women with ZIKV-infection and the correlation with their pregnancy outcomes.

## Introduction

Zika virus (ZIKV), a mosquito-borne flavivirus, is a clinically important pathogen. The outbreak of ZIKV in Brazil has created public health of international concern in 2015–2016 [1]. Despite mild clinical symptoms, ZIKV infection in pregnant women may cause a broad spectrum of abnormal pregnancy outcomes including intrauterine growth restriction (IUGR), microcephaly, miscarriage, and stillbirth [1–3]. The presence of ZIKV in placenta indicates its potential transmission from mother to embryo [1].

The pathogenesis of ZIKV in the placenta has been suggested to mediate the adverse pregnancy outcomes [4–6]. Experimental studies reveal that ZIKV causes placental damage and dysfunction [7, 8]. Notably, placental insufficiency or dysfunction, a clinical presentation of poor blood flow in the placenta, may lead to IUGR and pregnancy loss [9]. The placenta is a transient organ that facilitates the maternal-fetal exchange of gases, nutrients, hormones, and waste products through the blood-placental barrier (BPB). In addition, it provides a physical and immunological barrier against pathogen transmission from the mother to embryo [10]. The presence of pathogens in the intervillous maternal blood space may permit *in utero* viral transmission. However, to establish infection in the placenta and/or fetal organs, pathogens including viruses need to breach the BPB [11]. Thus, maintaining the placental function is critical for normal fetal development and preventing disease transmission.

In the context of pregnancy, folic acid (FA) supplementation is recommended for preventing neural tube defects in infants [12]. Notably, the coverage of FA supplementation among pregnant women in southern Brazil in 2013 was only 54.2% [13]. FA, a water-soluble B-vitamin, plays an important role in amino acid metabolism, cellular homeostasis, and DNA synthesis [14]. FA also plays an important role in maintaining vascular endothelial function by repressing nitric oxide production and homocysteine levels [15]. Moreover, its anti-inflammatory effect prevents bacteria-associated fetal growth restriction and abnormal pregnancy in mice [16]. Although the detail mechanisms remain unclear, FA signal transduction has various implications for feto-placental development and functions [17]. Mice deficient in folate receptor-α (FRα) are defective in early embryonic development [18]. Maternal exposure to FA antagonists increases the risk of placenta-mediated adverse pregnancy outcomes, including placental abruption, fetal growth restriction, and fetal death [19]. Furthermore, a high intake of supplemental FA greatly reduces the risk of spontaneous abortion [20]. Taking into account the role of FA in preventing poor pregnancy outcomes, we tested the potential effect of FA to alleviate the feto-placental pathology associated with ZIKV infection.

## Results

### Folic Acid (FA) displays antiviral effects against ZIKV infection in human umbilical vein endothelial cells (HUVECs)

**Fig 1:**
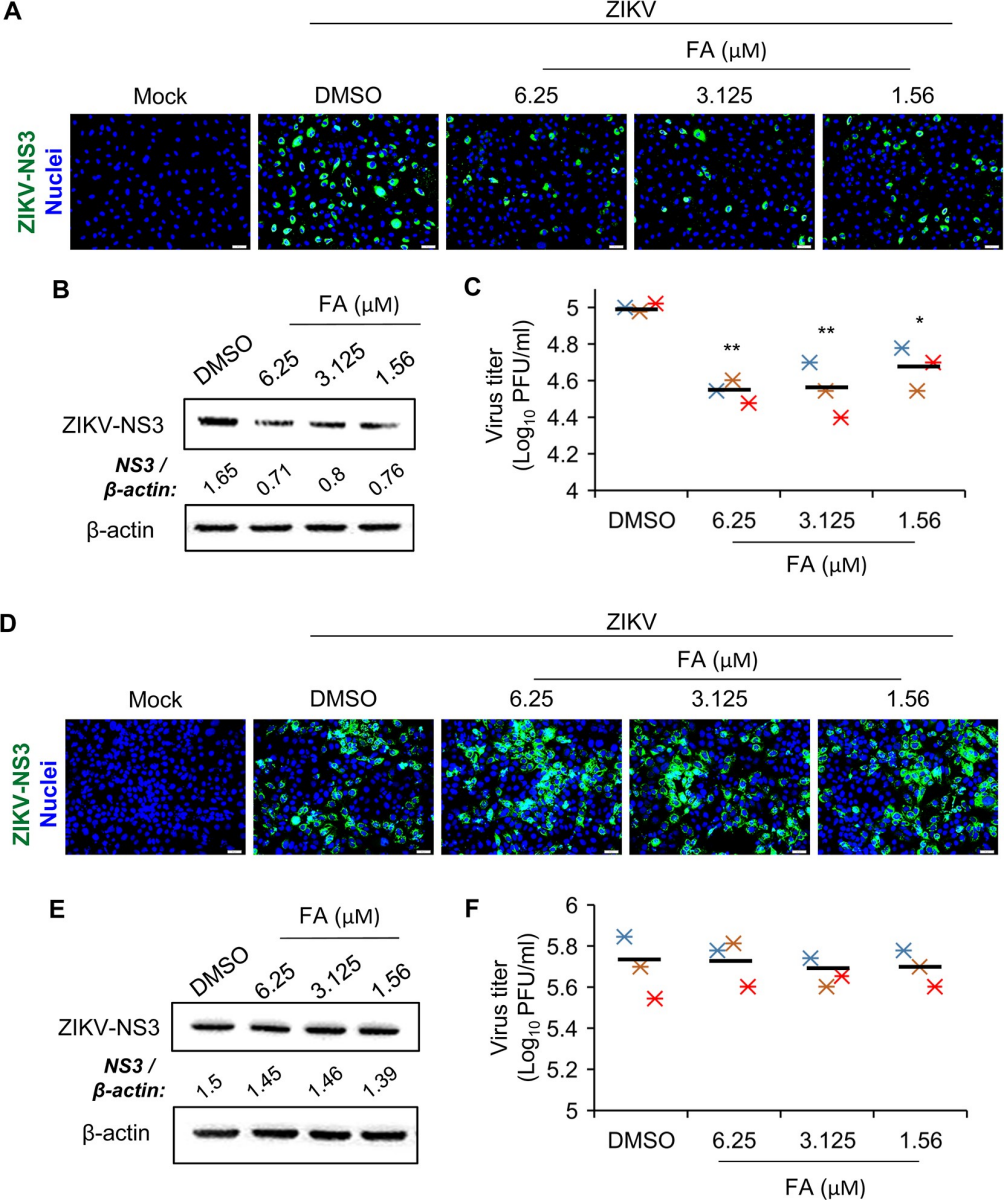
Folic acid (FA) displays antiviral effects against ZIKV infection in human umbilical vein endothelial cells (HUVECs). (A-C) HUVECs were pretreated with FA for 16 hr. Cells were infected with ZIKV in the presence of FA for 24 hr. Immunofluorescence microscopy was performed on cells immunostained for ZIKV-NS3 (green) and Hoechst for nuclei (blue) (A). Western blot analysis of the protein level of ZIKV-NS3 (B). Density ratios of respective protein and β-actin are shown in Western blot. Plaque-forming assay (PFA) of viral progeny production in culture supernatants (C). (D-F) Human trophoblast JEG-3 cells were pretreated with FA for 16 hr. Cells were infected with ZIKV in the presence of FA for 24 hr. Immunofluorescence microscopy was performed on cells immunostained for ZIKV-NS3 (green) and Hoechst for nuclei (blue) (D). Western blot analysis of the protein level of ZIKV-NS3 (E). Density ratios of ZIKV-NS3 and β-actin are shown in Western blot. Plaque-forming assay (PFA) of viral progeny production in culture supernatants (F). Data are mean (black bar) and individual values (n = 3 independent experiments). *P<0.05 and **P<0.01 by Kruskal-Wallis, Bonferroni post-hoc test.

The largest blood-placental barrier is in the labyrinth that consists of endothelial and trophoblast placental cells [11]. Therefore, we first evaluated antiviral activities of FA against ZIKV infection in HUVECs and human trophoblast placental JEG-3 cells. Longer duration of pretreatment of FA greatly repressed the expression of ZIKV-NS3 and virus progeny production in HUVECs, while the shorter pretreatment time did not affect ZIKV replication (16 vs. 2 hr; Fig 1A–1C and S1A–S1C Fig). In JEG-3 cells, FA did not display a significant anti-ZIKV activity regardless of the duration of pretreatment (16 vs. 2 hr; Fig 1D–1F and S1D–S1F Fig).

### Antiviral effect of FA against ZIKV infection is associated with folate receptor-α (FRα)-AMPK signal transduction

**Fig 2:**
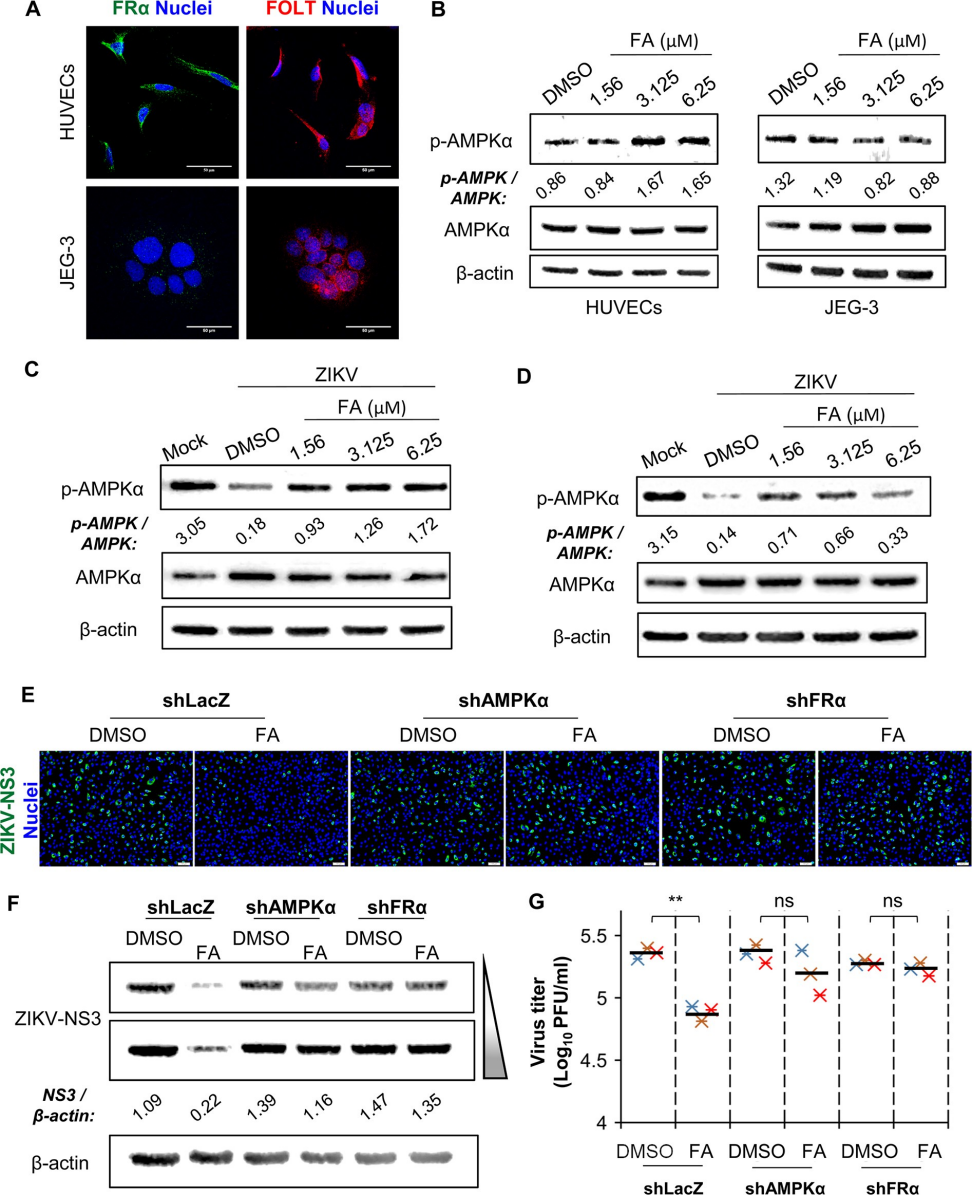
The antiviral effect of FA against ZIKV infection is associated with FRα-AMPK signal transduction. (A) Confocal images of HUVECs and JEG-3 cells immunostained for FRα (green), FOLT (red), and Hoechst for nuclei (blue). (B) Cells were treated with FA for 16 hr. Western blot analysis of the protein level of phospho-AMPK (p-AMPK), AMPK, and β-actin. Density ratios of p-AMPK and AMPK are shown in Western blot. (C-D) HUVECs (C) and JEG-3 cells (D) were treated with FA for 16 hr. Cells were infected with ZIKV in the presence of FA for 24 hr. Western blot analysis of the protein level of phospho-AMPK (p-AMPK), AMPK, and β-actin. Density ratios of p-AMPK and AMPK are shown in Western blot. (E-G) HUVECs were transfected with shRNA-targeting FRα (shFRα, TRCN0000372330), AMPKα (shAMPKα, TRCN0000000859), or control shRNA (shLacZ, TRCN0000072223) for 48 hr. Cells were treated with FA for 16 hr. Cells were infected with ZIKV in the presence of FA for 24 hr. Immunofluorescence microscopy was performed on cells immunostained for ZIKV-NS3 (green) and Hoechst for nuclei (blue) (E). Western blot analysis of the protein level of ZIKV-NS3 (F). Triangle indicates time of exposure. Density ratios of ZIKV-NS3 and β-actin are shown in Western blot. PFA of viral progeny production in culture supernatants (G). Data are mean (black bar) and individual values (n = 3 independent experiments). **P<0.01 by Mann-Whitney test. ns: not significant.

Folate receptor (FR) and transporter (FOLT) facilitate cellular uptake of FA. Notably, folate receptor-α (FRα) displays a high affinity for binding and transporting physiologic levels of FA into cells than any other FR isoforms and FOLT [21, 22]. FRα and FOLT were both expressed in HUVECs, while JEG-3 cells predominantly showed FOLT expression (Fig 2A). Low expression of FRα in JEG-3 cells also has been reported by a previous study [23]. Signal transduction of FA may implicate in adenosine-monophosphate activated protein kinase (AMPK) activation partly via an AMP-LKB1-dependent mechanism [24, 25]. We observed that FA treatments increased the level of phosphorylated-AMPK-α (p-AMPKα) in HUVECs, but not in JEG-3 cells (Fig 2B). Notably, ZIKV infection downregulated the expression of p-AMPKα in both HUVECs and JEG-3 cells, suggesting that ZIKV may inhibit AMPK activity (Fig 2C and 2D). FA treatments could greatly rescue the expression of p-AMPKα in ZIKV-infected HUVECS but displayed a minor effect in ZIKV-infected JEG-3 cells (Fig 2C and 2D). Interferon-α (IFN-α) has been shown to selectively sensitize HUVECs to double-stranded RNA-induced apoptosis that may implicate in restricting viral infections [26]. FA treatments in ZIKV-infected HUVECs increased the levels of IFN-α as compared to solvent-treated cells (S2A Fig). The effect of FA on the level of IFN-α was not significant in ZIKV-infected JEG-3 cells, presumably due to the low expression of FRα in this cell line (S2B Fig).

To further demonstrate the role of AMPK and FRα in mediating the anti-ZIKV property of FA in HUVECs, we transiently depleted the protein expressions by transfecting small hairpin RNA-targeting AMPKα (shAMPKα) or FRα (shFRα). Transfection with shRNAs decreased the protein expression of AMPKα or FRα in HUVECs (S2C and S2D Fig). Treatment with 6.25 μM of FA greatly reduced the level of ZIKV-NS3 and viral progeny production in control shLacZ-HUVECs (Fig 2E–2G). Notably, FA displayed a minor antiviral effect against ZIKV infection in shAMPKα- and shFRα-HUVECs (Fig 2E–2G). Thus, the inhibitory effect of FA against ZIKV infection was associated with FRα-AMPKα signaling.

### FA inhibits ZIKV-induced endothelial damage and reduces viral transmission in a cell culture model of blood-placental barrier (BPB)

**Fig 3:**
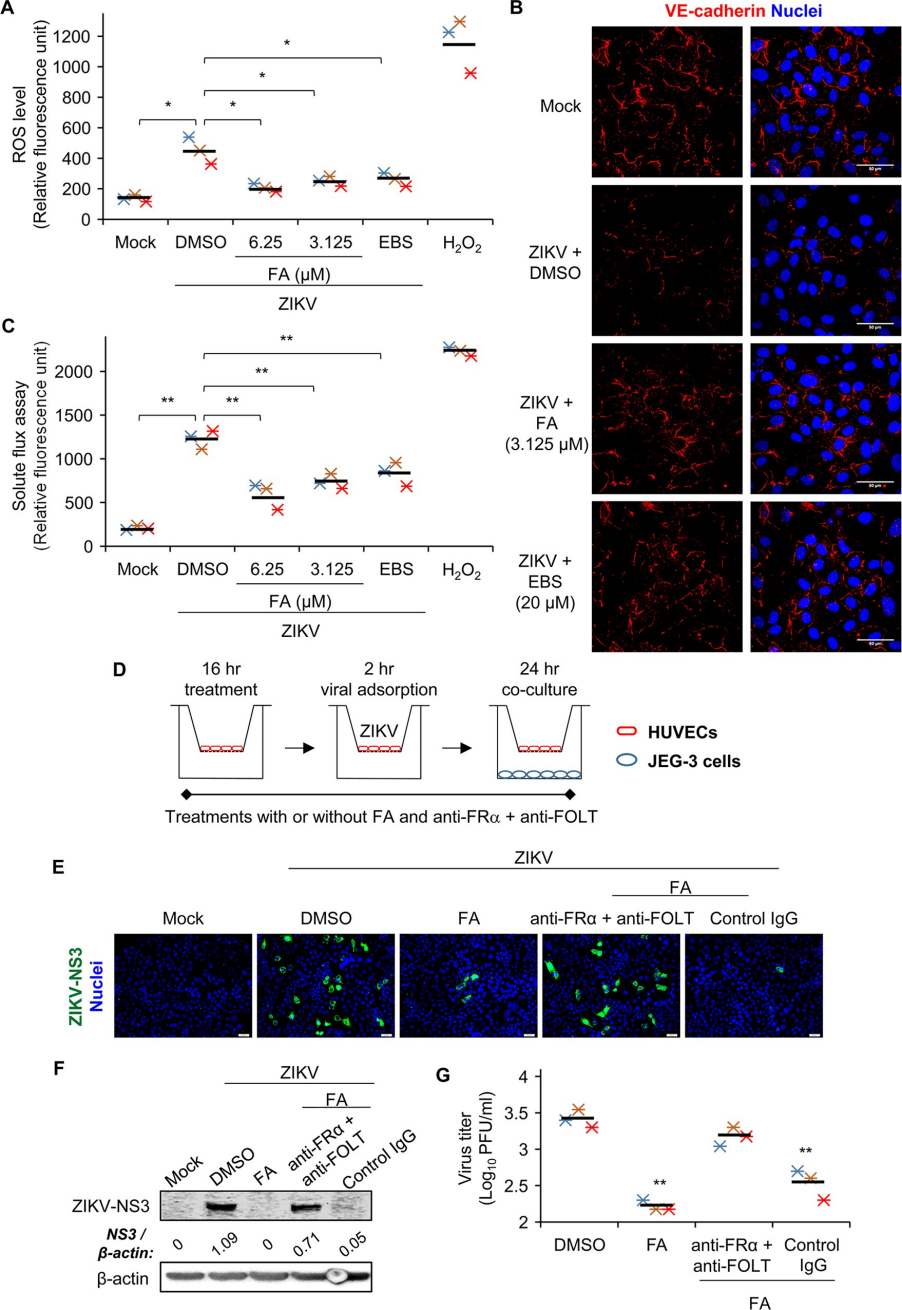
FA inhibits ZIKV-induced endothelial damage and reduces viral transmission in a cell culture model of blood-placental barrier (BPB). (A-C) HUVECs were treated with FA or 20 μM of antioxidant ebselen (EBS) for 16 hr. Cells were infected with ZIKV in the presence of FA, EBS or solvent (DMSO) for 24 hr. (A) Intracellular reactive oxygen species (ROS) assay. ROS levels were measured by the use of the OxiSelect intracellular ROS indicator. (B) Confocal images of cell surface expression of VE-cadherin. HUVECs were immunostained for VE-cadherin (red) and Hoechst for nuclei (blue). (C) Solute flux assay. The permeability of HUVECs was evaluated by the use of dextran-conjugated FITC. Fluorescence intensity of medium in the lower chamber was measured with a fluorescence microplate reader. H_2_O_2_ treatment was used as a positive control. (D) Schematic experimental design of a cell culture model of BPB. HUVECs, cultured in hanging inserts, were pre-treated with 3.125 μM of FA for 16 hr in the presence or absence of anti-folate receptor-α and anti-transporter antibodies (anti-FRα + anti-FOLT) or control IgG. Cells were adsorbed with ZIKV for 2 hr and co-cultured with JEG-3 cells for 24 hr. (E) Immunofluorescence microscopy was performed on JEG-3 cells immunostained for ZIKV-NS3 (green) and Hoechst for nuclei (blue). (F) Western blot analysis of the protein level of ZIKV-NS3 in JEG-3 cells. Density ratios of ZIKV-NS3 and β-actin are shown in Western blot. (G) PFA of viral progeny production in culture supernatants of JEG-3 cells. Data are mean (black bar) and individual values (n = 3 independent experiments). *P<0.05 and **P<0.01 by Kruskal-Wallis, Bonferroni post-hoc test.

To establish infection in the placenta and/or fetal organs, pathogens including viruses need to impair the BPB [11]. Vascular endothelial (VE)-cadherin, an adhesion molecule, plays a critical role in the formation of BPB [27]. Notably, reactive oxygen species (ROS) induces loss of surface expression of VE-cadherin. This, in turn, may weaken cell-cell junctions and damage endothelial permeability [28]. ZIKV infection significantly increased the level of intracellular ROS in HUVECs that could be reduced by the treatments of FA (Fig 3A). Moreover, FA rescued the cell surface expression of VE-cadherin in ZIKV-infected HUVECs (Fig 3B). FA also may inhibit ZIKV-associated endothelial hyperpermeability because treatments with FA greatly reduced the passage of macromolecule in solute flux assay (Fig 3C). In addition, treatment with antioxidant ebselen (EBS) that has been shown to reduce the levels of nitric oxide in sperms of ZIKV-infected mice [29] greatly repressed the level of ROS, recovered the expression of VE-cadherin, and improved cellular permeability in ZIKV-infected HUVECs (Fig 3A–3C). The disruption of cell-cell junction and endothelial permeability in HUVECs may not be limited to ZIKV. In agreement with the previous study [30], Japanese encephalitis virus (JEV) also downregulated the expression of VE-cadherin and impaired permeability of HUVECs (S3 Fig). On the contrary, dengue virus (DENV-2) infection did not affect the expression of VE-cadherin nor disrupt permeability of HUVECs (S3 Fig). Importantly, viral replication is essential to induce endothelial damage in HUVECs because UV-inactivated ZIKV did not adversely affect the expression of VE-cadherin and endothelial permeability (S3 Fig).

Next, we evaluated whether FA may prevent viral transmission in a cell culture model of BPB (Fig 3D). HUVECs were pre-treated with 3.125 μM of FA for 16 hr in the presence of anti-folate receptor-α and transporter (anti-FRα + anti-FOLT) antibodies or control IgG. ZIKV could be transmitted from HUVECs to JEG-3 cells, while FA treatment on HUVECs resulted in the lower viral burden in JEG-3 cells (Solvent vs. FA; Fig 3E–3G). In the presence of anti-FRα and anti-FOLT antibodies, FA treatment on ZIKV-infected HUVECs failed to reduce viral burden in JEG-3 cells as compared with the presence of IgG control antibody (anti-FRα+anti-FOLT vs. Control IgG; Fig 3E–3G). Overall, these data suggest that FA may inhibit ZIKV-induced endothelial damage and viral transmission in a cell culture model of BPB.

### FA alleviates feto-placental outcomes of ZIKV-infected pregnant AGB6 mice

**Fig 4:**
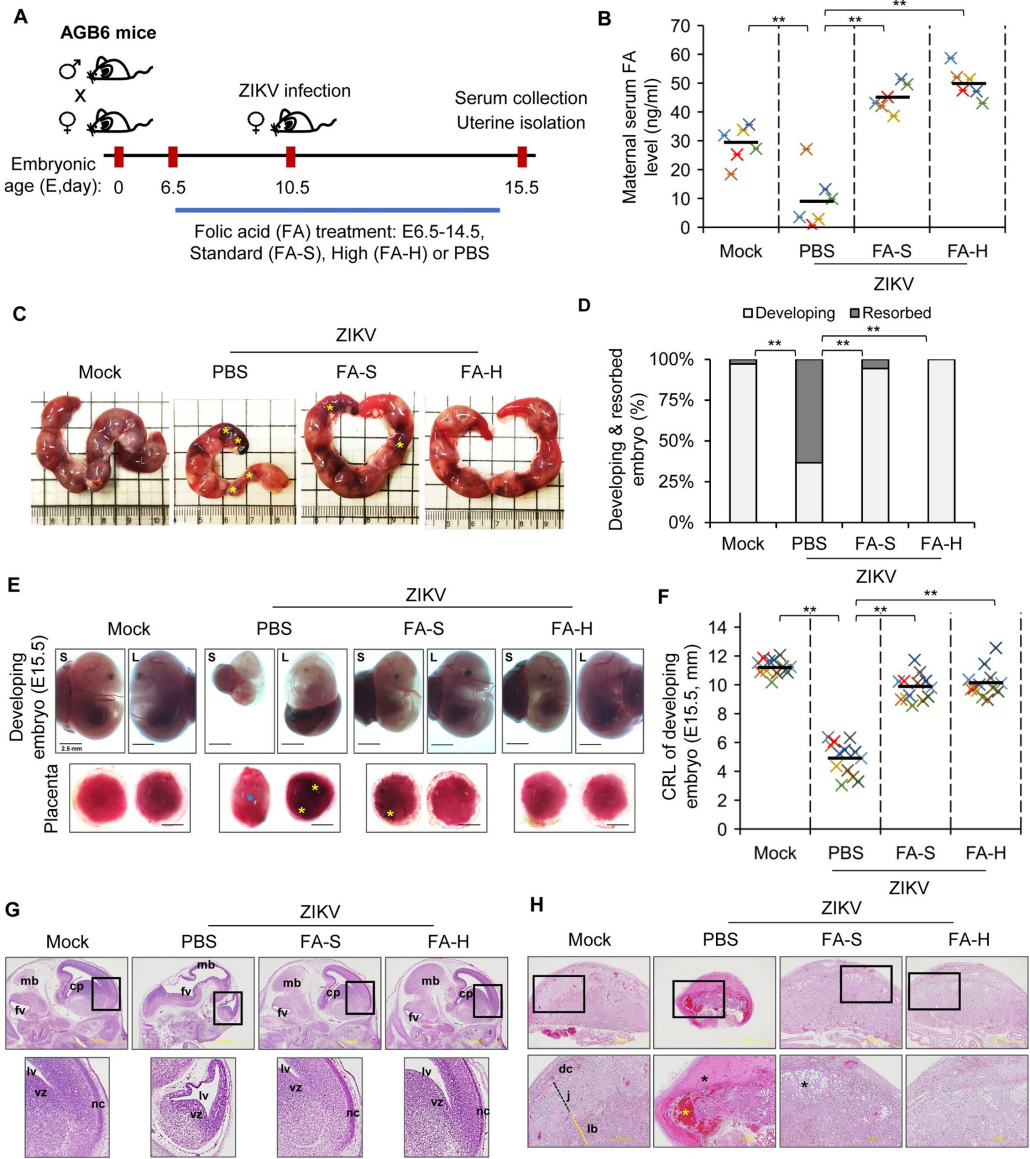
FA alleviates feto-placental outcomes of ZIKV-infected pregnant AGB6 mice. (A) Schematic experimental design. Interferon-α/β and -γ receptor-deficient AGB6 mice were mated overnight. Pregnant mice were treated with standard (FA-S, 0.164 mg/kg bw/mouse/oral/day) or high (FA-H, 0.328 mg/kg bw/mouse/oral/day) dose of folic acid (FA) at E6.5–14.5. PBS was used as solvent control. Pregnant mice were subcutaneously infected with ZIKV at E10.5. Uteri were isolated at E15.5. (B) Level of maternal serum FA of pregnant mice by ELISA. (C) Representative morphology of uterus. Fetal resorptions were indicated (yellow asterisk). (D) Percentage of developing and resorbed embryo. (E) Representative morphology of developing embryos and placentae. The largest (L) and smallest (S) embryos were presented. Placental edema (blue asterisk) and hemorrhage (yellow asterisk) were indicated. (F) Crown-rump length (CRL) of largest and smallest embryos. (G) Representative histological image of developing fetal brain (H&E staining). cp, choroid plexus; mb, midbrain; fv, fourth ventricle, lv, lateral ventricle; nc, neopallial cortex; vz, ventricular zone. (H) Representative histological image of the intact placenta (H&E staining) (40x magnification). dc, maternal decidua; j, junctional zone; lb, labyrinth. Necrotic lesion and hemorrhage were indicated (black and yellow asterisk, respectively). Data are mean (black bar) and individual values (n = 6 mice/group or 12 embryos/group). **P<0.01 by Kruskal-Wallis, Bonferroni post-hoc test.

We further tested whether FA could alleviate ZIKV-associated abnormal pregnancy outcomes in vivo. Immunocompromised pregnant mice have been used to model *in utero* transmission of ZIKV that show several pregnancy pathologies including placental damage, fetal resorption, and early postnatal death [8, 31]. To evaluate the beneficial effect of FA on ZIKV-associated IUGR and fetal demise, we used pregnant interferon-α/β and -γ receptor-deficient AGB6 mice. Pregnant mice were treated with standard-dose FA (FA-S, 0.164 mg FA/kg body weight (bw)/mouse/oral/day), high-dose FA (FA-H, 0.328 mg FA/kg bw/mouse/oral/day) or phosphate-buffered saline (PBS) as solvent control at embryonic day 6.5–14.5 (E6.5–14.5) as outlined in Fig 4A. To get a sufficient number of developing embryos and fetal demise for further analysis, pregnant mice were subcutaneously infected in the footpad with ZIKV at E10.5 [32]. Despite ZIKV-infected pregnant mice did not show any observable symptoms with undetectable ZIKV-NS3 expression in the brain (S4A Fig), the levels of serum FA were significantly lower than mock-infected pregnant mice (Fig 4B). Treatments with FA greatly increased the serum FA levels of ZIKV-infected pregnant mice (Fig 4B).

ZIKV infection in AGB6 mouse model caused abnormal pregnancies including uterine deformity and fetal demise (Fig 4C and S4B Fig). Fetal resorption was observed in more than 60% of the embryos of ZIKV-infected pregnant mice (Fig 4D). Remarkably, FA treatments greatly alleviated uterine abnormality and reduced the number of resorbed embryos in ZIKV-infected pregnant mice (Fig 4C and 4D). In addition, ZIKV-infected pregnant mice showed abnormal morphology of developing embryos and placentae at E15.5, including fetal growth restriction, abnormal placenta position and shape, edema, and hemorrhage (Fig 4E). Developing embryos of ZIKV-infected pregnant mice were significantly smaller than mock-infected embryos (Fig 4F). FA treatments in ZIKV-infected pregnant mice greatly improved feto-placental morphological features including normal placental position, a lower degree of placental hemorrhage, and larger size of developing embryos (Fig 4E and 4F). ZIKV infection affected overall fetal brain development, including small forebrain, absence of neopallial cortex, and abnormal fourth ventricle (Fig 4G). The levels of pro-inflammatory cytokines including interleukin 1β (IL-1β) and monocyte chemoattractant protein 1 (MCP-1) were upregulated in the ZIKV-infected fetal brains (S4C and S4D Fig). The embryos of ZIKV-infected pregnant mice receiving FA treatments showed improved fetal brain histology with a lower degree of inflammation (Fig 4G, S4C and S4D Fig). Moreover, ZIKV infection caused necrotic lesions in the maternal placental decidua and junctional zone, with a massive hemorrhage in the labyrinth (Fig 4H). ZIKV-infected pregnant mice receiving FA showed minor placental lesions in the junctional zone (Fig 4H).

### FA reduces the number of ZIKV-associated neonatal loss and improves the nutritional status of surviving pups

**Fig 5:**
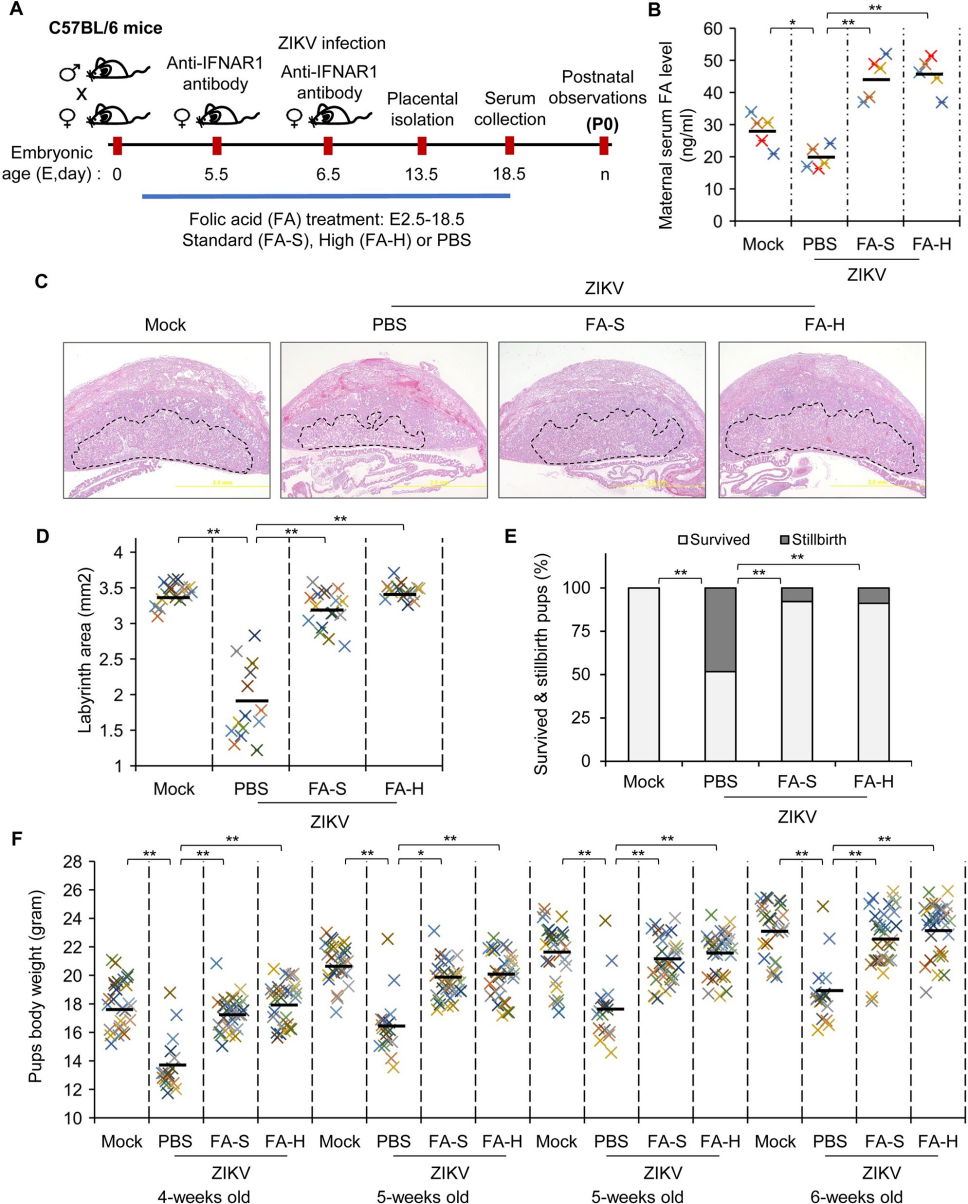
FA alleviates feto-placental outcomes of ZIKV-infected pregnant C57BL/6 mice. **(**A) Schematic experimental design. C57BL/6 mice were mated overnight. Pregnant mice were treated with FA-S (0.164 mg/kg bw/mouse/oral/day) or FA-H (0.328 mg/kg bw/mouse/oral/day) at E2.5–18.5. PBS was used as solvent control. Pregnant mice were treated with purified anti-mouse IFNAR1 antibody (0.5 mg/kg bw/mouse/intraperitoneal) at E5.5. Pregnant mice, at E6.5, were infected with ZIKV and subsequently treated with purified anti-mouse IFNAR1 antibody (0.5 mg/kg bw/mouse/intraperitoneal). Placentae were isolated at E13.5. Daily monitoring was performed to observe the term delivery and the death of the newborn within 12 hours (stillbirth). (B) Level of maternal serum FA. Maternal sera at E18.5 were collected for quantification of FA by ELISA. (C) Representative histological image (H&E staining) of placentae at E13.5. The labyrinth area was marked with a black line. (D) Labyrinth area of the placentae. (E) Percentage of survived and stillbirth pups. (F) The body weight of litters. Data are mean (black bar) and individual values (n = 5 mice/group, 13–17 histological images/group, or 17–31 pups/group). *P<0.05 and **P<0.01 by Kruskal-Wallis, Bonferroni post-hoc test.

To study the effect of FA on the number of neonatal death and postnatal nutritional status, we performed *in utero* transmission of ZIKV by the use of anti-interferon-α/β receptor 1 (IFNAR1) antibody-treated wild-type (WT) C57BL/6 pregnant mice (Fig 5A). Pregnant mice were treated with FA-S, FA-H or PBS at E2.5–18.5. IFNAR1 antibody was delivered to pregnant mice at E5.5 and E6.5, one day before and right after ZIKV infection at early placentation [33]. In this mouse model, ZIKV infection at E6 yields a higher viral load in the placenta than viral inoculation at E9 or E12 [34]. In this C57BL/6 mouse model, ZIKV infection caused a number of stillbirths and the low body weight of surviving pups [31]. In addition to postnatal observation, placentae were isolated at E13.5 for further analysis. First, we also evaluated the levels of maternal FA in ZIKV-infected pregnant mice. Consistent with AGB6 mouse model, ZIKV-infected C57BL/6 pregnant mice showed lower serum FA levels than mock pregnant mice at E18.5; FA treatments significantly increased the serum levels of FA in ZIKV-infected pregnant mice (Fig 5B). ZIKV infection has been shown to reduce the area of maternal-fetal exchange, the placental labyrinth [8]. FA treatments significantly alleviated the histopathology of ZIKV in terms of the size of the placental labyrinth (Fig 5C and 5D, S4E Fig). We observed 48% death of pups within 12 hr after term delivery in ZIKV-infected mice, whereas FA-treated mice had less than 10% stillbirths (Fig 5E, S4F and S4G Fig). Notably, ZIKV protein was detected in the brain of stillbirth pups (S4H Fig). To evaluate the nutritional status at a later age, we prospectively monitored the body weight of surviving pups in the weaning age up to 7-week-old. Although the surviving pups of ZIKV-infected dams gained weight throughout the monitoring period, their body weight was significantly lower than that of mock-infected mice (Fig 5F). Importantly, the body weight was higher for surviving pups of ZIKV-infected dams receiving FA than solvent treatment (Fig 5F). Taken together, these data suggest that FA alleviates pregnancy abnormality of ZIKV-infected pregnant mice.

### FA limits ZIKV replication in the placenta and improves the prognostic profile of placental dysfunction

**Fig 6:**
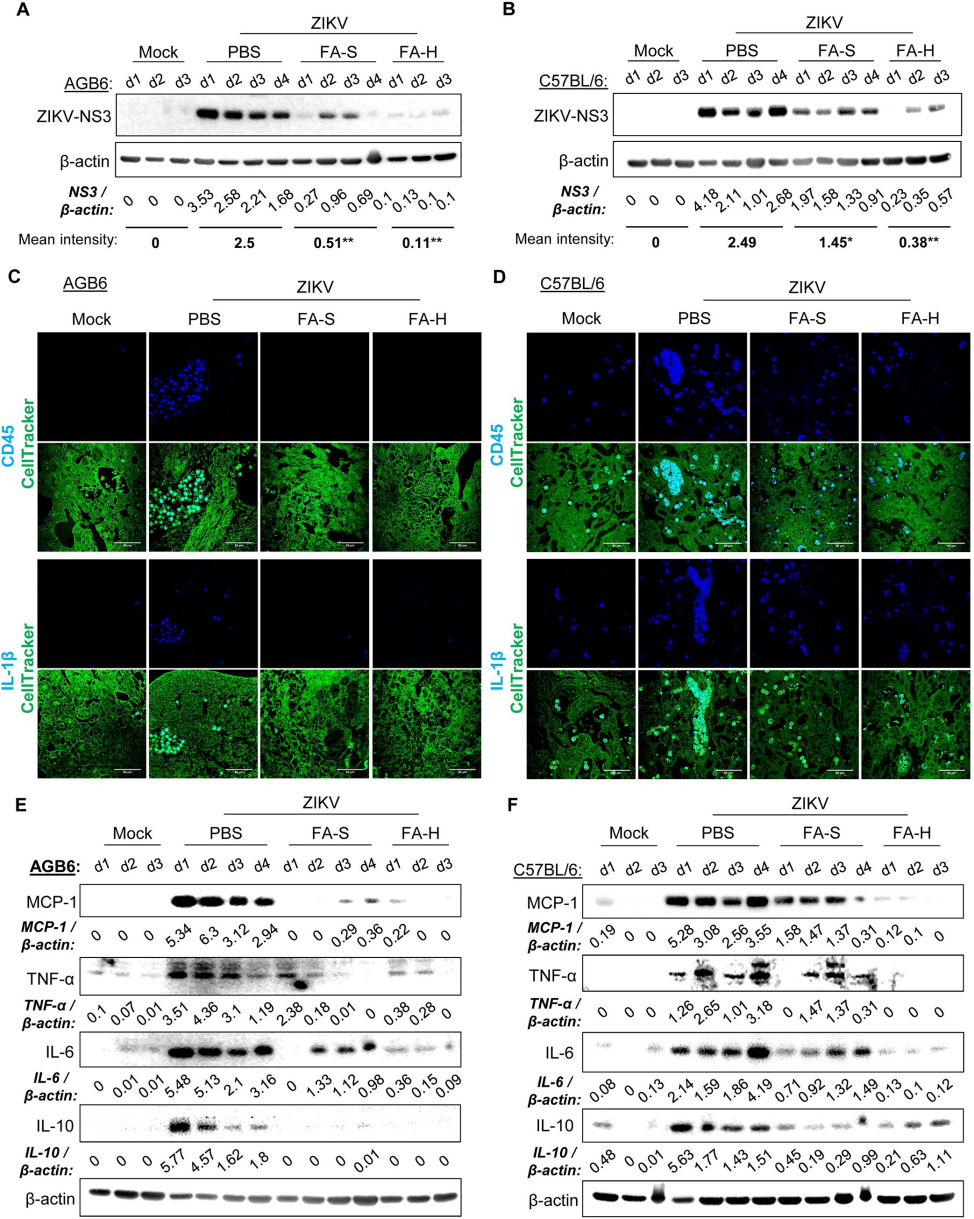
FA limits ZIKV replication and alleviates ZIKV-induced inflammatory response in the placenta. **(**A-B) ZIKV burden in the placenta of AGB6 (A, E15.5) and IFNAR1 antibody-treated C57BL/6 (B, E13.5) mice. Western blot analysis of protein level of ZIKV-NS3. (C-D) Infiltration of leukocyte in the placenta of AGB6 (C) and IFNAR1 antibody-treated C57BL/6 (D) mice. Representative confocal images of placental sections immunostained for CD45 (blue, upper panels), IL-1β (blue, lower panels), and CellTracker for cytoplasm (green). (E-F) Western blot analysis of protein levels of inflammatory cytokines in the placenta of AGB6 (E) and IFNAR1 antibody-treated C57BL/6 (F) mice, β-actin for loading control. Density ratios of respective protein and β-actin are shown in Western blot. Samples are pooled placental tissue lysates of 3–4 representative pregnant mice (d1-d4). *P<0.05 and **P<0.01 by Kruskal-Wallis, Bonferroni post-hoc test.

Since FA alleviated ZIKV-induced pathological features in the placenta, we expected a lower viral burden in the placenta of FA-treated pregnant mice. Indeed, FA treatments greatly reduced the expression of ZIKV-NS3 protein in the placenta of infected pregnant mice (Fig 6A and 6B), with no significant effect on maternal ZIKV viremia level (S5A and S5B Fig). Notably, FA treatments had no effect on the median survival time of ZIKV-infected non-pregnant mice (S5C and S5D Fig). These data suggest that FA may display tissue-specific effects. FRα abundantly expressed in the placenta and developing embryo; however, the expression is minimal in other tissues or organs [22, 35]. Without FA treatments, robust expression of ZIKV-NS3 was noted in trophoblast giant cells of the junctional zone, villous trophoblast cells of the labyrinth, and trophoblast cells surrounding necrotic lesions (S5E and S5F Fig). Furthermore, FA greatly reduced the levels of ZIKV RNA in the placentae as well as developing embryos (S6 Fig).

It is well-known that viral infection triggers inflammatory responses. The elevated pro-inflammatory response is attributed to poor placental function and pregnancy outcomes [36]. Leukocyte infiltration and elevated levels of pro-inflammatory cytokines impair vascular endothelial growth in a model of placental insufficiency [36]. Notably, FA reduces the levels of pro-inflammatory cytokines that are associated with BPB dysfunction, including IL-1β, MCP-1, and TNF-α [37–39]. We observed that ZIKV-infected pregnant mice showed a systemic inflammation, as indicated by a significant increase of serum C-reactive protein (CRP) levels (S7A and S7B Fig). FA treatments could significantly reduce the serum levels of CRP in ZIKV-infected pregnant mice (S7A and S7B Fig). ZIKV infection also caused infiltration of CD45 and IL-1β–positive cells in the placental labyrinth, which could be inhibited by FA treatments (Fig 6C and 6D). Moreover, FA repressed ZIKV-induced inflammatory response in the placenta, including MCP-1, TNF-α, IL-6, and IL-10 levels (Fig 6E and 6F, S7C and S7D Fig).

**Fig 7:**
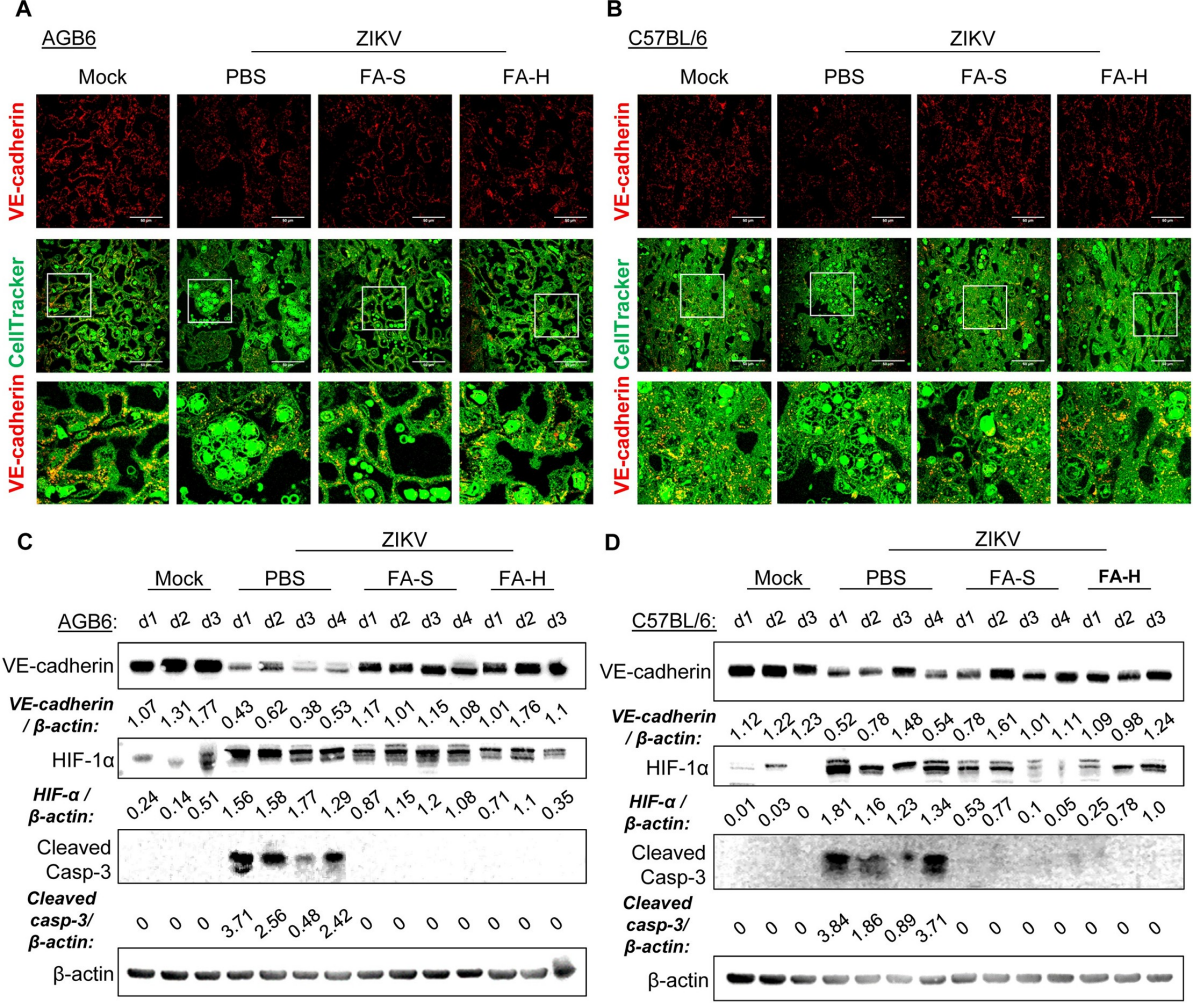
FA improves the prognostic profile of placental dysfunction. (A-B) Representative confocal images of placental sections of AGB6 mice (A) and IFNAR1 antibody-treated C57BL/6 (B) Placenta immunostained for VE-cadherin (red) and CellTracker for cytoplasm (green). (C-D) Western blot analysis of protein level of VE-cadherin, HIF-1α, cleaved caspase-3, and β-actin for loading control of placental AGB6 (C) and IFNAR1 antibody-treated C57BL/6 (D) mice. Samples are pooled placental tissue lysates of 3–4 representative pregnant mice (d1-d4).

ZIKV infection downregulated the placental expression of VE-cadherin (Fig 7A–7D, S7C and S7D Fig). Notably, FA treatments rescued the placental expression of VE-cadherin (Fig 7A–7D, S7C and S7D Fig). Because increased levels of inflammatory cytokines and poor blood flow are associated with utero-placental hypoxia [40], we further evaluated the expression of hypoxia-inducible factor-1α (HIF-1α) in the placenta. ZIKV infection upregulated HIF-1α expression, an adverse response that could be alleviated by FA treatments (Fig 7C and 7D, S7C and S7D Fig). HIF-1 has a role in hypoxia inducing apoptosis [41]. Consistently, the active form of caspase-3 in ZIKV-infected placentae was greatly increased and could be suppressed by FA treatments (Fig 7C and 7D, S7C and S7D Fig). Overall, our data suggest that FA treatment in ZIKV-infected pregnant mice could reduce viral burden in the placenta and improve the prognostic profile of placental insufficiency.

## Discussion

The role of FA in viral infection is still limited. Our study reveals the beneficial effect of FA in preventing *in utero* ZIKV transmission and improving feto-placental disease prognosis. Direct infection of BPB and/or pathogen-induced pro-inflammatory response may weaken the integrity of the maternal-fetal interface to permit *in utero* viral transmission [42]. A recent study shows that activation of AMPK inhibits ZIKV replication in HUVECs by inducing antiviral response, inhibiting the inflammatory response and/or viral-induced glycolysis that could be cell- or tissue-specific [43, 44]. In this study, we report that FA recovered AMPKα activity, increased the level of interferon-α, and inhibited viral replication in ZIKV-infected HUVECs. Interestingly, FA remained potent in reducing viral load and alleviating pathological features in the placenta of interferon-α/β and -γ receptor-knockout AGB6 mice, suggesting interferon-independent modes of therapeutic action of FA in animals. Studies have documented the role of FA in suppressing endothelial oxidative stress and placental inflammatory response, which in turn alleviates the disease pathology [15, 16]. Moreover, AMPK activation is not only necessary for the placental differentiation and growth but also regulating the inflammatory response in the placenta [45]. Intriguingly, both antiviral and anti-inflammatory effects of FA may play a role in limiting ZIKV transmission through maternal-fetal interface. Further studies are required to elucidate the role of FA in viral-induced glycolysis.

FRα is expressed abundantly and consistently in the syncytiotrophoblast of BPB during pregnancy [22]. Despite structural differences between human and mouse BPB, they share similar functions, proteomic profile, and physiological conditions [33]. The largest BPB is in the labyrinth that consists of syncytiotrophoblast-cytotrophoblast in humans and two syncytiotrophoblast layers-cytotrophoblast in rodents, respectively. The second maternal-fetal interface is extravillous trophoblast in humans or trophoblast giant cells in rodents [11, 33]. The syncytiotrophoblast is a unique trophoblast that has more than one nucleus (multinucleate) and forms a continuous syncytial layer as a result of cell-cell fusion. In humans, the syncytiotrophoblast forms the outer surface of the villi and makes direct contact with maternal blood. The syncytiotrophoblast also plays a role in waste product removal and synthesis of pregnancy hormones [33].

The underlying mechanism of ZIKV-induced low level of maternal FA in our study is unclear. Intriguingly, ZIKV infection may reduce the dietary intake of FA due to loss of appetite and/or adversely affect FA transport and pool in the liver. Recent observations in humans indicate that ZIKV infection causes abnormal liver function and severe liver injury [46, 47]. A case-control study in Brazil suggested that maternal use of FA might not be associated with ZIKV-related microcephaly [48]. However, this study did not evaluate the serum level of maternal FA that could be confounded by ZIKV infection and/or smoking during pregnancy. Pregnant women who smoked had significantly lower serum maternal FA levels than non-smokers, although their FA intake was relatively the same [49]. Notably, the risk of ZIKV-associated microcephaly is 3-fold-increased in smoking pregnant women [48]. These studies may argue the use of a higher dose of FA supplement for smoking pregnant women with ZIKV infection. Moreover, a cross-sectional study in Southern Brazil in 2013 reported the use of FA during pregnancy was considerably poor (54.2%) [13], suggesting that the FA supplementation program needs to be revitalized.

As calculated by a published method [50], the equivalent dose of 0.164 mg FA/kg/day (FA-S) for mice is 0.0133 mg/kg/day for humans; thus, for a 60-kg person, the dose would be about 800 μg/day. The US Preventive Services Task Force recommends that women of childbearing age take 400–800 μg FA/day to prevent neural tube defects in infants [12]. A high dose of FA up to 5 mg/day has been proven safe in healthy women [51]; however, the beneficial effect of a high FA dose remains controversial. In preeclamptic pregnant women, daily supplementation with 1 and 5 mg FA does not significantly differ in affecting disease outcomes [52]. Importantly, in the presence of vitamin B12 deficiency, ≥ 5 mg of FA intake may lead to adverse clinical presentations including anemia and cognitive impairment [53].

Overall, our findings reveal the beneficial effect of FA supplementation to alleviate the adverse pregnancy outcomes associated with ZIKV infection in immunocompromised AGB6 and WT C57BL/6 mouse models. Nutritional surveillance to evaluate maternal FA status in areas with active ZIKV transmission is required to revitalize FA supplementation program among women at childbearing for preventing ZIKV-associated abnormal pregnancy.

## Materials and methods

### Ethics statement

The mouse experiments were conducted according to the guideline outlined by Council of Agriculture Executive Yuan, Republic of China. This animal protocol was approved by the Academia Sinica Institutional Animal Care and Use Committee (Protocol no. 16-06-966) and were performed in accordance with the guidelines. Infection was performed in mice under isoflurane anesthesia and all efforts were made to minimize animal suffering.

### Virus strain and propagation

ZIKV epidemic PRVABC59 strain (2015 Puerto Rico strain, Genbank accession: KU501215) and DENV-2 PL046 strain (Genbank accession: AJ968413.1) was kindly provided by the Centers for Disease Control, Taiwan. The neurovirulent RP-9 strain of JEV [54] was used for in vitro study. ZIKV was propagated in C6/36 mosquito cells (ATCC: CRL-1660) and the level of infectious virus particles was measured by plaque-forming assay (PFU/ml) in Vero cells (ATCC: CRL-1587) as described [55].

### Cell culture studies

Primary human umbilical vein endothelial cells (HUVECs, ATCC: CRL-1730) were grown in 1% gelatin-coated plate and cultured in M199 (Gibco) containing 20% fetal bovine serum (FBS), 50 μg/ml of endothelial cell growth supplement (Sigma, E2759), and 100 μg/ml of heparin. Passage number 3–7 of HUVECs were used for cell culture experiments. Human choriocarcinoma trophoblast placental JEG-3 (ATCC: HTB-36) were cultured in MEM (Gibco) containing 10% FBS. Cells pretreated for 2 or 16 hr with the indicated concentrations of FA were adsorbed with ZIKV (multiplicity of infection [MOI] = 1) for 2 hr in the presence or absence of FA, washed to remove unbound viruses, and incubated for 24 hr in the presence or absence of FA. The antiviral effect of FA was evaluated by immunofluorescence, western blot, and plaque-forming assays.

The level of intracellular ROS was determined by using the OxiSelect Intracellular ROS (Cell Biolabs, STA-342) assay kit. Briefly, HUVECs were incubated with 1x DCFH-DA (diluted in HBSS without phenol red) for 45 minutes at 37°C. Treatment with 50 μM of H_2_O_2_ or 20 μM of antioxidant ebselen (EBS, Cayman Chemical, CAS 60940-34-3) treatment was used as control experiments. Mock infected cells were used as baseline control. Fluorescence intensity was determined by using a fluorescence reader (Spectramax, Molecular Devices). For solute flux assay, HUVECs were grown in 1% gelatin-coated 24-well hanging inserts (Millipore). Cells were treated with solvent or FA at the indicated doses for 16 hr. Cells were infected with ZIKV (MOI = 1) for 24 hr in the absence or presence of FA. The apical chamber was replenished with culture medium containing FITC-CM-Dextran (70-kDA, Sigma, 53471) and the lower chamber was loaded HBSS with phenol red. Cells were incubated for 40 minutes. Fluorescence intensity of medium in the lower chamber was measured with a fluorescence microplate reader at 480/530 nm (excitation/emission). Quantitative assay for interferon-α in the cell culture media was performed according to the manufacturer’s protocol (VeriKine Human IFN alpha ELISA kit, PBL Assay Science, 41100). Briefly, 100 μl of cell culture medium was used for a sandwich immunoassay. The absorbance of samples was determined by use of an ELISA reader (Molecular Devices) at 450 nm. The concentration of the sample was extrapolated from the standard curve.

For the cell culture model of the blood-placental barrier, HUVECS were grown in 1% gelatin-coated 24-well hanging inserts (Millipore). Cells were treated with 3.125 μm of FA for 16 hr in the presence or absence of 2 μg of antibodies against folate receptor-α (FRα) and folate transporter (FOLT) or control IgG. Cells were adsorbed with ZIKV (MOI = 1) for 2 hr and co-cultured with JEG-3 cells for 24 hr. JEG-3 cells and culture supernatants were used for immunofluorescence, western blot, and plaque-forming assays.

The loss-of-function study was performed in HUVECs by transfecting pLKO.1 vector plasmid which carries a short hairpin RNA (shRNA) targeting the human FRα (TRCN0000060347, TRCN0000372330), human AMPKα (TRCN0000000859, TRCN0000000861), or LacZ (TRCN0000072223). pLKO.1-shRNAs were obtained from the Taiwan National RNAi Core Facility. Transfection was performed as described [56] with modification. Briefly, 0.8 μg of plasmid diluted in Opti-MEM (Gibco) was transfected into HUVECs by use of Lipofectamine 2000 (Invitrogen). Cells were incubated for 4 hr. Subsequently, the complete culture medium was added and the cells were incubated for 48 hr. Knockdown efficiency was checked at 48 hr after transfection. Transfected cells were used to evaluate the antiviral effect of FA.

Antibodies for flavivirus NS3 [57], AMPKα (Cell Signaling, 2532S), phospho-AMPKα (GeneTex, GTX52341; Cell Signaling, 2535S), FRα (Thermo, PA5-42004), and FOLT (LSBio, LS-C139788) were used in cell culture studies.

### Mouse study

The minimal sample size of each group was calculated by the use of a published method [58]. Mice under isoflurane anesthesia were infected and all efforts were made to minimize animal suffering. Seven- to 8-week-old male and female AGB6 mice with interferon-α/β and -γ receptor-knockout (National Health Research Institutes, Taiwan) or wild-type C57BL/6JNarl (WT-C57BL/6, National Laboratory Animal Center, Taiwan) mice were mated overnight. The day 0.5 of embryonic age (E0.5) was defined as the first observation of a vaginal plug. To study the effect of FA (Sigma, F7876) on feto-placental outcomes, each pregnant mouse was orally given 0.164 mg FA/kg body weight/day (standard dose of FA [FA-S]), 0.328 mg FA/kg body weight/day (high dose of FA [FA-H]), or solvent control (phosphate-buffered saline [PBS]) by the use of feeding needle (stainless steel, olive tip). FA treatments were given to pregnant AGB6 and WT-C57BL/6 mice at E6.5–14.5 and E2.5–18.5, respectively. Pregnant AGB6 mice were subcutaneously infected in the footpad with 1×10^2^ plaque-forming units (PFU) of ZIKV per mouse. The culture supernatant of C6/36 mosquito cells was used for mock infection. Maternal blood sera and uteri of pregnant AGB6 mice were collected at E15.5. Pregnant WT mice were treated with purified anti-mouse IFNAR1 antibody (0.5 mg/kg body weight/mouse/intraperitoneal) at E5.5. Pregnant mice were intravenously injected in the tail vein with 1×10^6^ PFU of ZIKV per mouse, then treated with anti-mouse IFNAR1 antibody (0.5 mg/kg body weight/mouse/intraperitoneal) at E6.5. Placentae and maternal sera were collected at E13.5 and E18.5, respectively. Mice were monitored daily to observe the term delivery and death of newborns within 12 hr (stillbirth). To evaluate the antiviral effect of FA against ZIKV infection in vivo, groups of 7- to 8-week-old non-pregnant AGB6 mice were subcutaneously infected in the footpad with 1×10^2^ PFU of ZIKV per mouse. Immediately after infection, mice were treated with FA-S, FA-H, or PBS orally. Thereafter, mice received the same treatments up to day 10 after infection. The mice were checked daily for mortality. Serum was collected on day 3 after infection by phlebotomy from a facial vein, then the viral load was determined by plaque-forming assay.

### Quantification of FA and CRP levels, histology, immunohistochemistry, western blot analysis, and quantitative RT-PCR

Maternal sera underwent ELISA for the quantification of FA (Cell Biolabs, MET-5068) and CRP (Abcam, ab157712). The serum levels of FA and CRP were determined by comparison with the respective standard curves. Mouse hearts were perfused with cold PBS to minimize maternal blood accumulation in the uterus. Uteri were collected immediately after death in cold PBS. Embryos and placentae were carefully isolated from uteri by using a dissecting microscope (Olympus SZ61). Crown-rump length (CRL) of embryos was measured under an Olympus SZ61 microscope. Embryos and placentae were fixed overnight in Bouin’s solution (Sigma, HT10132), then washed with 50% alcohol for tissue processing and embedding. Histology involved hematoxylin-eosin staining of 3-μm-thick embryo and placenta sections observed under a bright-field Olympus BX51 microscope. For immunohistochemistry, embryo and placenta sections were immunostained with anti-flavivirus NS3[57], anti-CD45 (Novusbio, NB100-77417), anti-IL-1β (Abcam, ab2105), or anti-VE-cadherin (Abcam, ab33168) antibodies. Hoechst (Invitrogen, H21492) and CellTracker Green (Invitrogen, c2925) were used to stain the nucleus and cytoplasm, respectively. The confocal analysis involved the confocal laser scanning microscope ZEISS LSM 700. Placentae and brain tissues were digested in cold RIPA lysis buffer, sonicated at 21% amplitude for 1 min, then incubated on ice for 10 min. Homogenate was centrifuged at 10,000 rpm for 10 min at 4°C and supernatants were collected for western blot analysis to evaluate the expression of flavivirus NS3 or E [29, 57], MCP-1 (Invitrogen, 41–0900), TNF-α (Abcam, ab6671), IL-6 (Invitrogen, mp5-20f3), IL-10 (Abcam, ab33471), VE-cadherin (Abcam, ab33168), HIF-1α (Novus Biologicals, nb100-479) and cleaved caspase-3 (Cell Signaling, 9664s). Total RNA was extracted from the brain of developing embryos or placentae by using TRIzol (Thermo, 15596018) and Direct-zol RNA MiniPrep purification kit (Zymo, R2051). cDNA was reverse-transcribed from 100 ng of RNA with random hexamers by using SuperScript II RT kit (Life Technologies, 18064022). PCR involved use of the PowerUp SYBR Green master CYBR Green Gene Expression kit (Thermo, A25743) with the primers for ZIKV envelope (5′-TTGGTCATGATACTGCTGATTGC-3′ and 5′-CCYTCCACRAAGTCYCTATTGC-3’). The ZIKV RNA level were calculated by the ^−Δ^*CT* value and normalized to that of GAPDH for relative quantification.

### Statistical analysis

Data were compared by Kruskal-Wallis Bonferroni post-hoc or Mann-Whitney test. The statistical tests were two-tailed and significance was set at *P* < 0.05. For immunoblotting, the band density was quantified by using ImageJ (US National Institutes of Health)

## Supporting information

S1 Fig Antiviral effect of FA in vitro. (A-C) HUVECs were pretreated with FA for 2 hr. Cells were infected with ZIKV in the presence or absence of FA for 24 hr. Immunofluorescence microscopy was performed on cells immunostained for ZIKV-NS3 (green) and Hoechst for nuclei (blue) (A). Western blot analysis of the protein level of ZIKV-NS3 (B). Plaque-forming assay (PFA) of viral progeny production in culture supernatants (C). (D-F) JEG-3 cells were pretreated with FA for 2 hr. Cells were infected with ZIKV in the presence or absence of FA for 24 hr. Immunofluorescence microscopy was performed on cells immunostained for ZIKV-NS3 (green) and Hoechst for nuclei (blue) (D). Western blot analysis of the protein level of ZIKV-NS3 (E). PFA of viral progeny production in culture supernatants (F). Density ratios of ZIKV-NS3 and β-actin are shown in Western blot. Data are mean (black bar) and individual values (n = 3 independent experiments).
(TIF)

S2 Fig Effect of FA on the level of IFN-α in vitro and knockdown efficiency of shRNA. (A-B) Levels of IFN-α in cell culture supernatants. HUVECs (A) and JEG-3 cells (B) were pretreated with FA for 16 hr. Cells were infected with ZIKV in the presence or absence of FA for 24 hr. IFN-α levels were measured by the use of the VeriKine human IFN-α Elisa kit. Data are mean (black bar) and individual values (n = 3 independent experiments). (C-D) Knockdown efficiency of shRNA. HUVECs were transfected with shRNA-targeting FRα (shFRα), AMPKα (shAMPKα), or control shRNA (shLacZ). (C) Representative confocal images of cells immunostained for FRα (green) and Hoechst for nuclei (blue). (D) Western blot analysis of protein level of AMPKα. Density ratios of AMPKα and β-actin are shown in Western blot.
(TIF)

S3 Fig Effect of UV-inactivated ZIKV, dengue virus (DENV), and Japanese encephalitis virus (JEV) infection on the expression of VE-cadherin and endothelial permeability of HUVECs. Cells were infected with UV-inactivated ZIKV, dengue virus (DENV-2), or Japanese encephalitis virus (JEV) at the indicated multiplicity of infection (MOI) for 24 hr. (A). Confocal images of cell surface expression of VE-cadherin. HUVECs were immunostained for VE-cadherin (red) and Hoechst for nuclei (blue). (B) Solute flux assay. The permeability of HUVECs was evaluated by the use of dextran-conjugated FITC. Fluorescence intensity of medium in the lower chamber was measured with a fluorescence microplate reader. Data are mean (black bar) and individual values (n = 3 independent experiments). **P<0.01 compared with mock by Kruskal-Wallis, Bonferroni post-hoc test.
(TIF)

S4 Fig Viral burden in the maternal brain, fetal resorption, neuroinflammation, placental histology, and stillbirth. (A) Expression of ZIKV NS3 in the maternal brain of pregnant AGB6 mice (E15.5). Placental lysate of ZIKV-infected mouse receiving PBS treatment was used as a positive control. (B) Representative morphology of fetal resorption in ZIKV-infected pregnant AGB6 mice (p, residual placenta; f, resorbed fetus). (C) Western blot analysis of protein levels of inflammatory cytokines in the fetal brain of AGB6, β-actin for loading control. Samples are pooled developing fetal brain lysates of 3–4 representative pregnant mice (d1-d4). (D) Representative Western blot analysis of protein levels of inflammatory cytokines in the fetal brain of AGB6, β-actin for loading control. Samples are individual developing fetal brains (e: an individual fetal brain lysate) of 1 representative pregnant mouse (d1). (E) Representative histological image (H&E staining) of placentae at E13.5 of three pregnant mice (d1-d3). The labyrinth area was marked with a black line. **(**F-G) Representative morphology of surviving pups of mock-infected C57BL/6 mice (F) and stillbirth of ZIKV-infected C57BL/6 mice (G). (H) Western blot analysis of ZIKV-E in pooled brain lysates of stillbirth pups of C57BL/6 mouse, β-actin for loading control.
(TIF)

S5 Fig Viremia level of pregnant mice, mouse survival, and ZIKV distribution in the placenta. **(**A) Viremia levels of ZIKV-infected pregnant AGB6 mice on day 3 after infection. (B) Viremia levels of ZIKV-infected pregnant C57BL/6 mice on day 2 after infection. (C-D) Non-pregnant AGB6 mice were subcutaneously infected in the footpad with 1×10^2^ PFU of ZIKV per mouse. Mice were treated with FA-S, FA-H, or PBS on day 0–10 after infection. Mouse survival presented as a percentage of survival. The median survival time (T_50_) is presented (C). Survival curves were compared by Log-rank test. Viremia level of mice on day 3 after infection (D). (E-F) ZIKV distribution in the placenta of AGB6 (in C, E15.5) and C57BL/6 (in D, E13.5) mice. Representative immunohistochemistry placental images with hematoxylin counterstaining. Signals of ZIKV-NS3 were developed by DAB chromogenic reaction.
(TIF)

S6 Fig ZIKV RNA level in the placenta and fetal brain. (A-B) Relative quantitative analysis of the ZIKV RNA levels in the individual placentae of AGB6 (A, E15.5) and C57BL/6 (B, E13.5) mice (n = 11–16 cDNA/group). (C-D) Relative quantitative analysis of ZIKV RNA levels in the fetal brain of AGB6 (C, E15.5) and C57BL/6 (D, E13.5) mice [n = pooled cDNA of 3–6 pregnant mice/ group (C) or 11–16 individual cDNA/group (D)]. Data are mean (black bar) and individual values. *P<0.05 and **P<0.01 by Kruskal-Wallis, Bonferroni post-hoc test.
(TIF)

S7 Fig Maternal serum CRP and Western blot analysis of individual placentae. (A-B) The level of maternal serum CRP of ZIKV-infected pregnant AGB6 mice (A, E15.5) and ZIKV-infected pregnant C57BL/6 mice (B, E18.5). Data are mean (black bar) and individual values (n = 6 or 5 mice/group). **P<0.01 by Kruskal-Wallis, Bonferroni post-hoc test. (C-D) Western blot analysis of protein levels of inflammatory cytokines, VE-cadherin, HIF-1α, cleaved caspase-3 in the placenta of AGB6 (C) and C57BL/6 (D) mice, β-actin for loading control. Density ratios of respective protein and β-actin are shown in Western blot. Samples are individual placental tissue lysates (p1-p9) of 1 representative pregnant mouse (d1).
(TIF)
