# Correction: Preventive effects of folic acid on Zika virus-associated poor pregnancy outcomes in immunocompromised mice

**DOI:** 10.1371/journal.ppat.1014363

**Published:** 2026-06-26

**Authors:** Yogy Simanjuntak, Hui-Ying Ko, Yi-Ling Lee, Guann-Yi Yu, Yi-Ling Lin

After this article [1] was published, concerns were raised with Fig 7, S1 Fig and S4 Fig in S1 File. Specifically:

- In Fig 7B, the Mock VE-cadherin panel appears to overlap with the Fig 7B ZIKV FA-S VE-cadherin panel.
- In Fig 7B, the Mock CellTracker panel appears to overlap with the Fig 7B ZIKV FA-S CellTracker panel.
- In S1E Fig in S1 File, beta-actin (lanes 2–5) appears similar to S4H Fig in S1 File beta-actin (lanes 1–4).

Corresponding author YLL stated that the confocal immunofluorescence images for the Mock group of C57BL/6 mice in Fig 7B are incorrect. A correct version of Fig 7 is provided here where the three Mock panels in Fig 7B have been replaced with the correct panels from the original experiments, and the white rectangles in the CellTracker FA-S and FA-H panels in Fig 7B have been moved. The underlying data for Fig 7 is provided in S1 File.

Corresponding author YLL provided the underlying blots for S1 Fig and S4 Fig in S1 File. The *PLOS Pathogens* Editors therefore consider this concern resolved.

The raw data underlying Figs 1-5, S1-S3 Figs in S1 File and S5-S7 Figs in S2 File are missing from the list of Supporting Information. The authors have provided the data as Supporting Information S1-S2 Files. With this correction, all relevant data are now provided.

**Fig 7:**
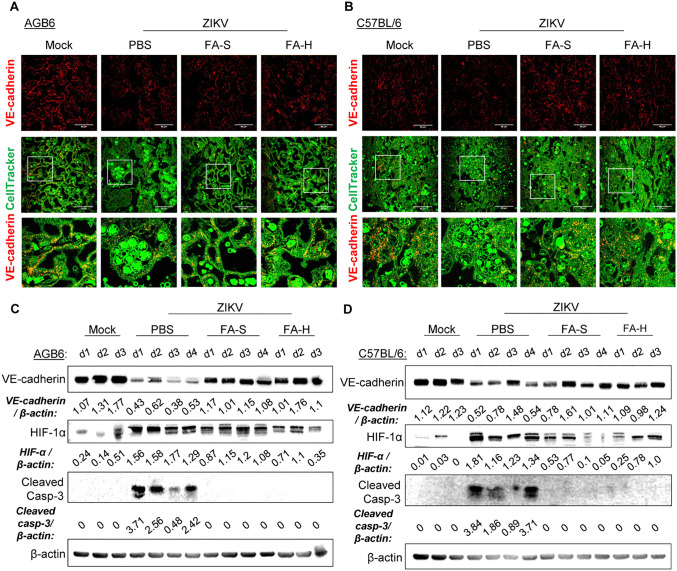
FA improves the prognostic profile of placental dysfunction. **(A-B)** Representative confocal images of placental sections of AGB6 mice (A) and IFNAR1 antibody-treated C57BL/6 **(B)** Placenta immunostained for VE-cadherin (red) and CellTracker for cytoplasm (green). **(C-D)** Western blot analysis of protein level of VE-cadherin, HIF-1α, cleaved caspase-3, and β-actin for loading control of placental AGB6 (C) and IFNAR1 antibody-treated C57BL/6 (D) mice. Samples are pooled placental tissue lysates of 3–4 representative pregnant mice (d1-d4).

## Supporting information

S1 File Underlying data in support of Figs 2A – 2G, 5B-5F, 7A-7D, S1A-S1F, S4A-and S4H. (ZIP)

S2 File Underlying data in support of Figs 1C, 1F, 3A, 3C, 3G, 4B, 4D, 4F, S2A, S2B, S3B, S5A, -S5D, S6A-S6D, S7A-S7B. (ZIP)

## References

[1] Simanjuntak Y, Ko H-Y, Lee Y-L, Yu G-Y, Lin Y-L. Preventive effects of folic acid on Zika virus-associated poor pregnancy outcomes in immunocompromised mice. PLoS Pathog. 2020;16(5):e1008521. doi: 10.1371/journal.ppat.1008521 32392268 PMC7241851

