# Supplementary figures and images for: Preventive effects of folic acid on Zika virus-associated poor pregnancy outcomes in immunocompromised mice
Source: PLoS Pathog. 2020 May 11;16(5):e1008521. doi: 10.1371/journal.ppat.1008521 (PMC7241851; doi:10.1371/journal.ppat.1008521)

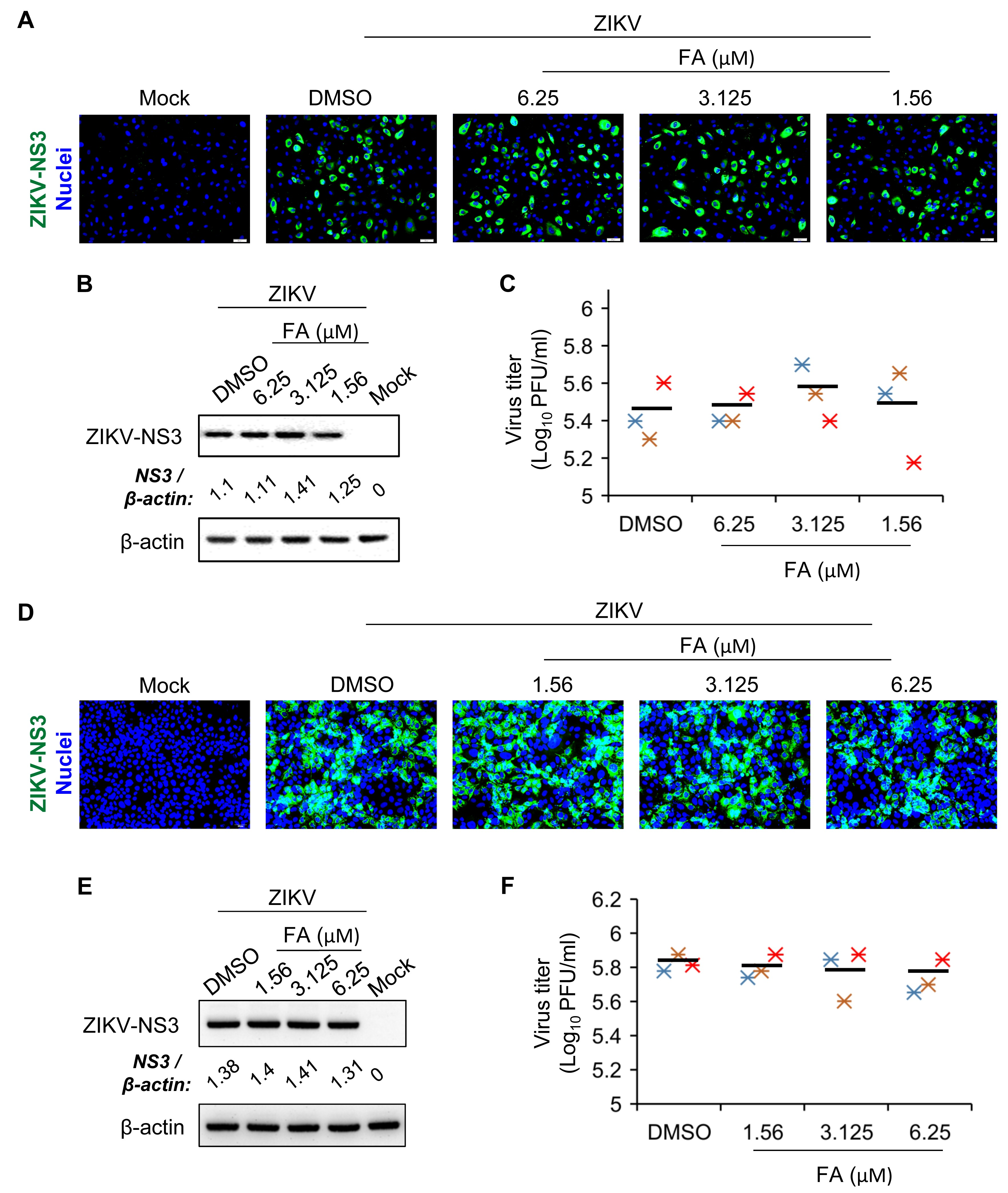

Supplement: S1 Fig — (A-C) HUVECs were pretreated with FA for 2 hr. Cells were infected with ZIKV in the presence or absence of FA for 24 hr. Immunofluorescence microscopy was performed on cells immunostained for ZIKV-NS3 (green) and Hoechst for nuclei (blue) (A). Western blot analysis of the protein level of ZIKV-NS3 (B). Plaque-forming assay (PFA) of viral progeny production in culture supernatants (C). (D-F) JEG-3 cells were pretreated with FA for 2 hr. Cells were infected with ZIKV in the presence or absence of FA for 24 hr. Immunofluorescence microscopy was performed on cells immunostained for ZIKV-NS3 (green) and Hoechst for nuclei (blue) (D). Western blot analysis of the protein level of ZIKV-NS3 (E). PFA of viral progeny production in culture supernatants (F). Density ratios of ZIKV-NS3 and β-actin are shown in Western blot. Data are mean (black bar) and individual values (n = 3 independent experiments). (TIF) [file ppat.1008521.s001.tif]

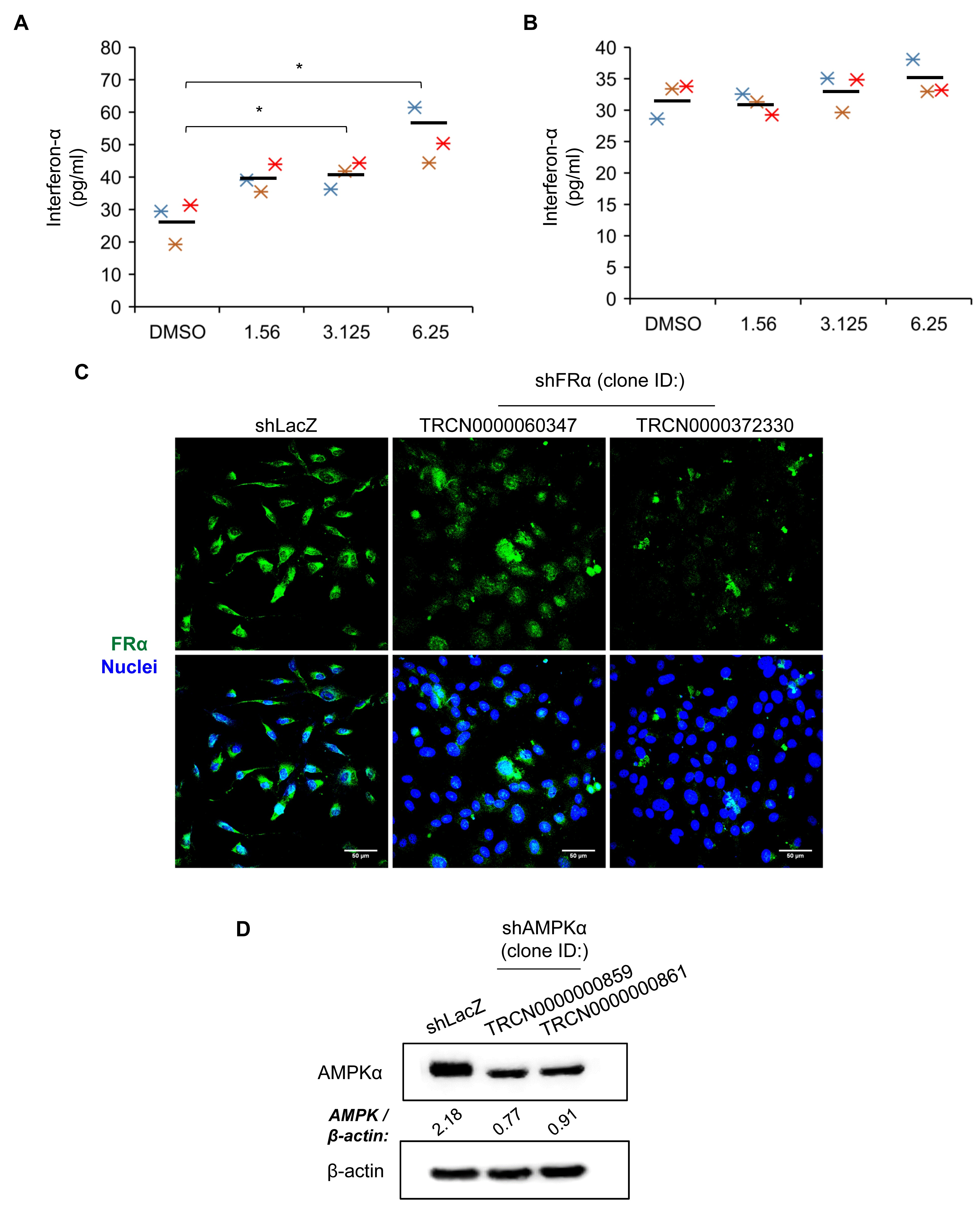

Supplement: S2 Fig — (A-B) Levels of IFN-α in cell culture supernatants. HUVECs (A) and JEG-3 cells (B) were pretreated with FA for 16 hr. Cells were infected with ZIKV in the presence or absence of FA for 24 hr. IFN-α levels were measured by the use of the VeriKine human IFN-α Elisa kit. Data are mean (black bar) and individual values (n = 3 independent experiments). (C-D) Knockdown efficiency of shRNA. HUVECs were transfected with shRNA-targeting FRα (shFRα), AMPKα (shAMPKα), or control shRNA (shLacZ). (C) Representative confocal images of cells immunostained for FRα (green) and Hoechst for nuclei (blue). (D) Western blot analysis of protein level of AMPKα. Density ratios of AMPKα and β-actin are shown in Western blot. (TIF) [file ppat.1008521.s002.tif]

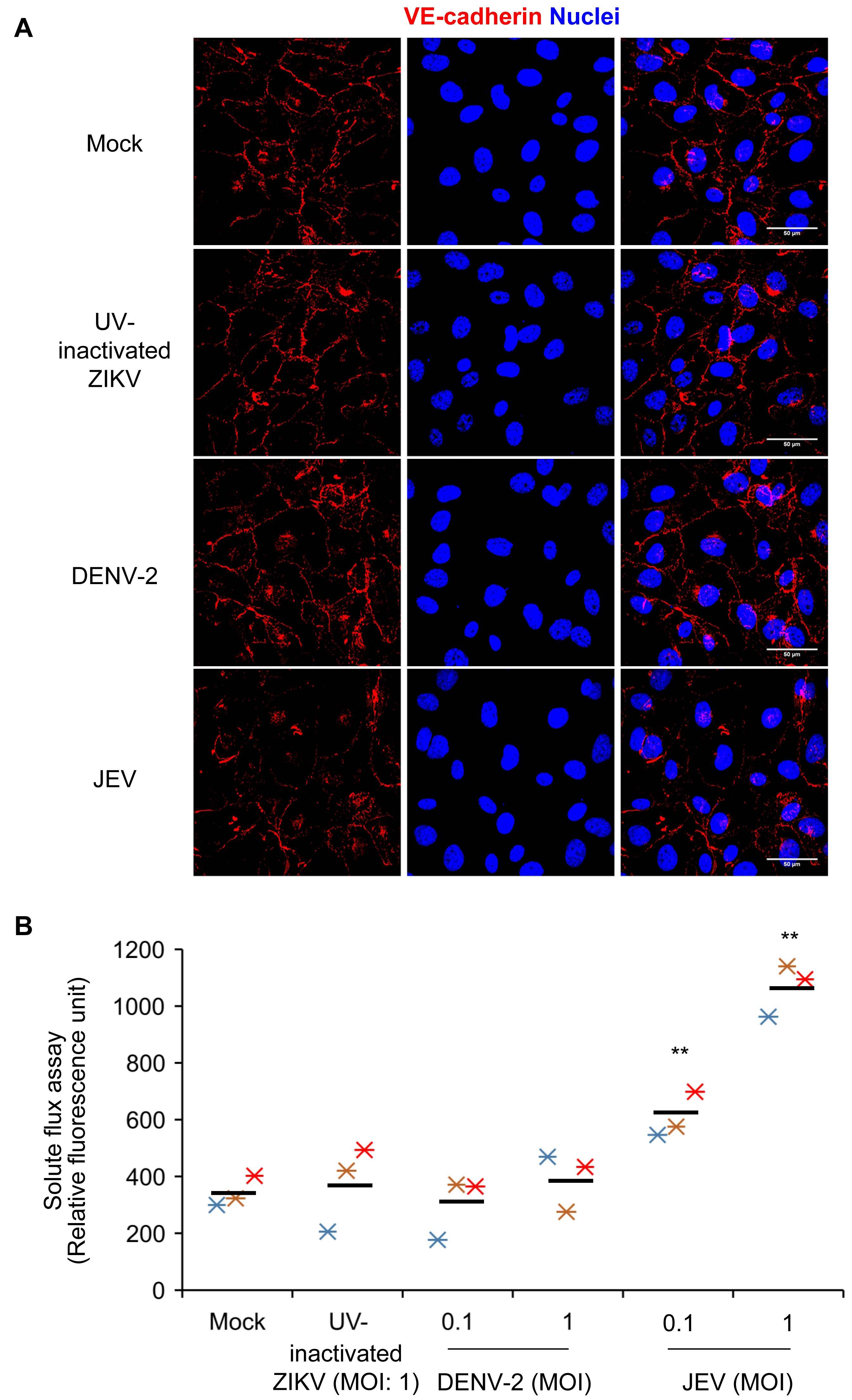

Supplement: S3 Fig — Cells were infected with UV-inactivated ZIKV, dengue virus (DENV-2), or Japanese encephalitis virus (JEV) at the indicated multiplicity of infection (MOI) for 24 hr. (A). Confocal images of cell surface expression of VE-cadherin. HUVECs were immunostained for VE-cadherin (red) and Hoechst for nuclei (blue). (B) Solute flux assay. The permeability of HUVECs was evaluated by the use of dextran-conjugated FITC. Fluorescence intensity of medium in the lower chamber was measured with a fluorescence microplate reader. Data are mean (black bar) and individual values (n = 3 independent experiments). **P<0.01 compared with mock by Kruskal-Wallis, Bonferroni post-hoc test. (TIF) [file ppat.1008521.s003.tif]

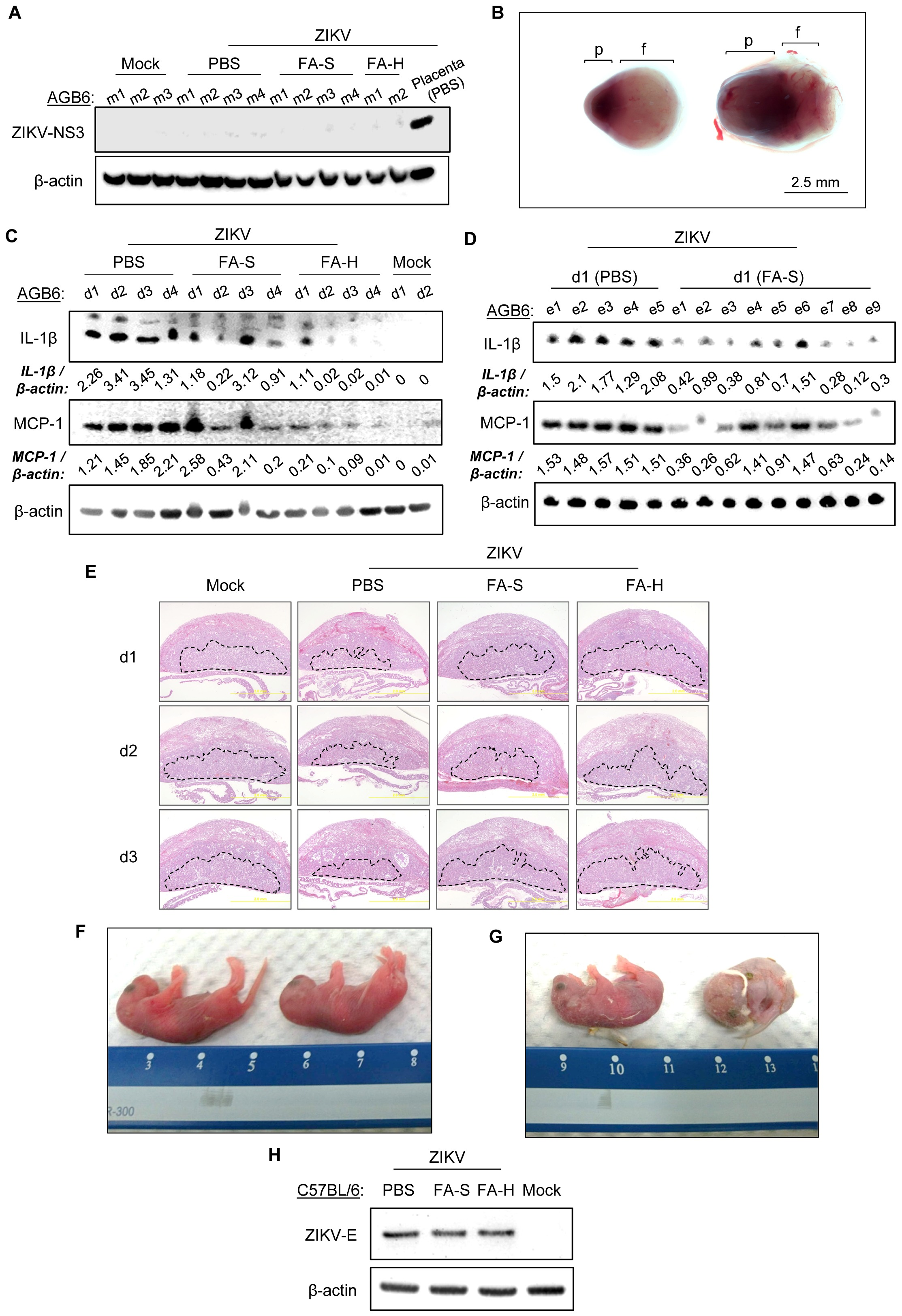

Supplement: S4 Fig — (A) Expression of ZIKV NS3 in the maternal brain of pregnant AGB6 mice (E15.5). Placental lysate of ZIKV-infected mouse receiving PBS treatment was used as a positive control. (B) Representative morphology of fetal resorption in ZIKV-infected pregnant AGB6 mice (p, residual placenta; f, resorbed fetus). (C) Western blot analysis of protein levels of inflammatory cytokines in the fetal brain of AGB6, β-actin for loading control. Samples are pooled developing fetal brain lysates of 3–4 representative pregnant mice (d1-d4). (D) Representative Western blot analysis of protein levels of inflammatory cytokines in the fetal brain of AGB6, β-actin for loading control. Samples are individual developing fetal brains (e: an individual fetal brain lysate) of 1 representative pregnant mouse (d1). (E) Representative histological image (H&E staining) of placentae at E13.5 of three pregnant mice (d1-d3). The labyrinth area was marked with a black line. (F-G) Representative morphology of surviving pups of mock-infected C57BL/6 mice (F) and stillbirth of ZIKV-infected C57BL/6 mice (G). (H) Western blot analysis of ZIKV-E in pooled brain lysates of stillbirth pups of C57BL/6 mouse, β-actin for loading control. (TIF) [file ppat.1008521.s004.tif]

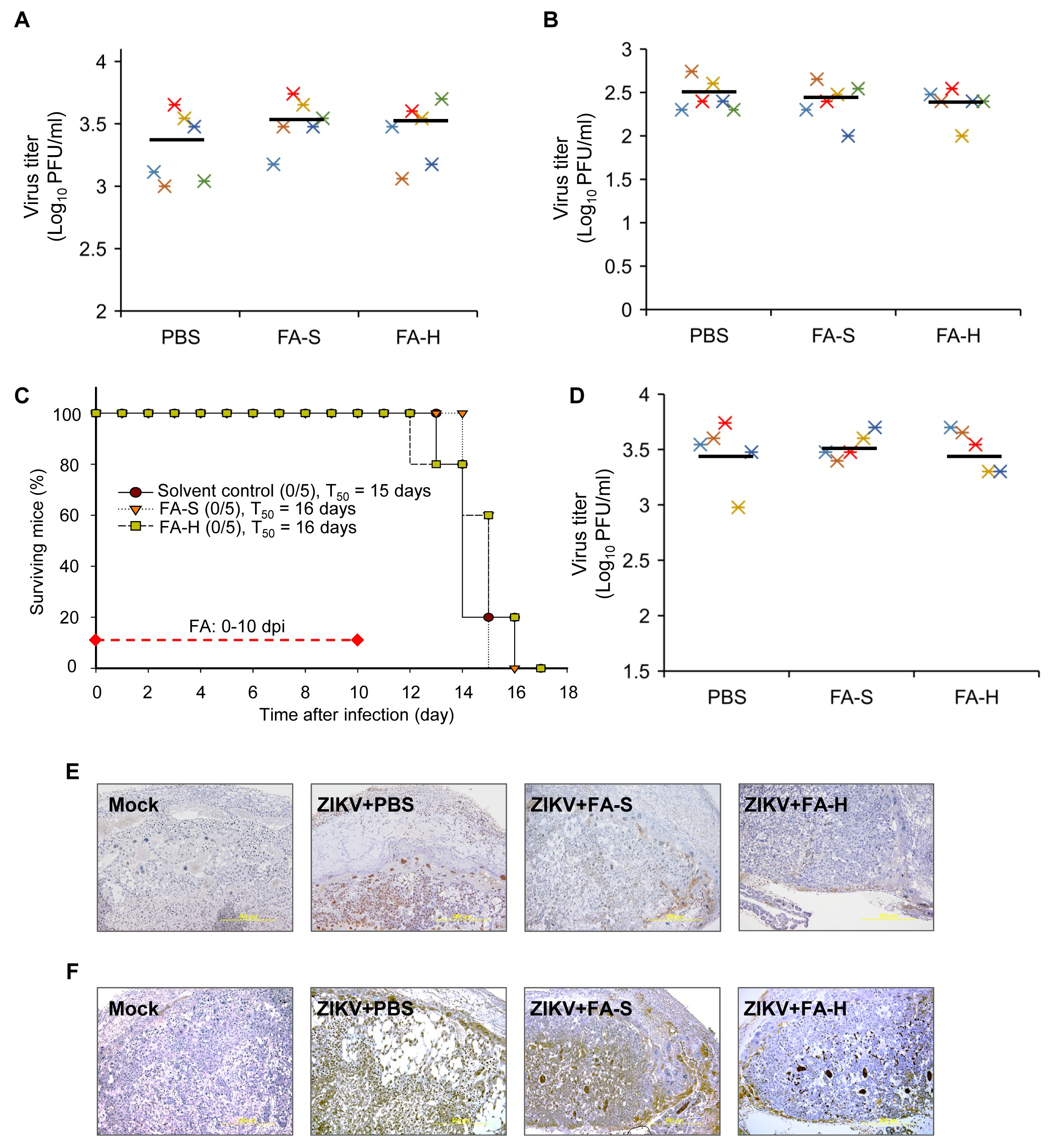

Supplement: S5 Fig — (A) Viremia levels of ZIKV-infected pregnant AGB6 mice on day 3 after infection. (B) Viremia levels of ZIKV-infected pregnant C57BL/6 mice on day 2 after infection. (C-D) Non-pregnant AGB6 mice were subcutaneously infected in the footpad with 1×10^2^ PFU of ZIKV per mouse. Mice were treated with FA-S, FA-H, or PBS on day 0–10 after infection. Mouse survival presented as a percentage of survival. The median survival time (T50) is presented (C). Survival curves were compared by Log-rank test. Viremia level of mice on day 3 after infection (D). (E-F) ZIKV distribution in the placenta of AGB6 (in C, E15.5) and C57BL/6 (in D, E13.5) mice. Representative immunohistochemistry placental images with hematoxylin counterstaining. Signals of ZIKV-NS3 were developed by DAB chromogenic reaction. (TIF) [file ppat.1008521.s005.tif]

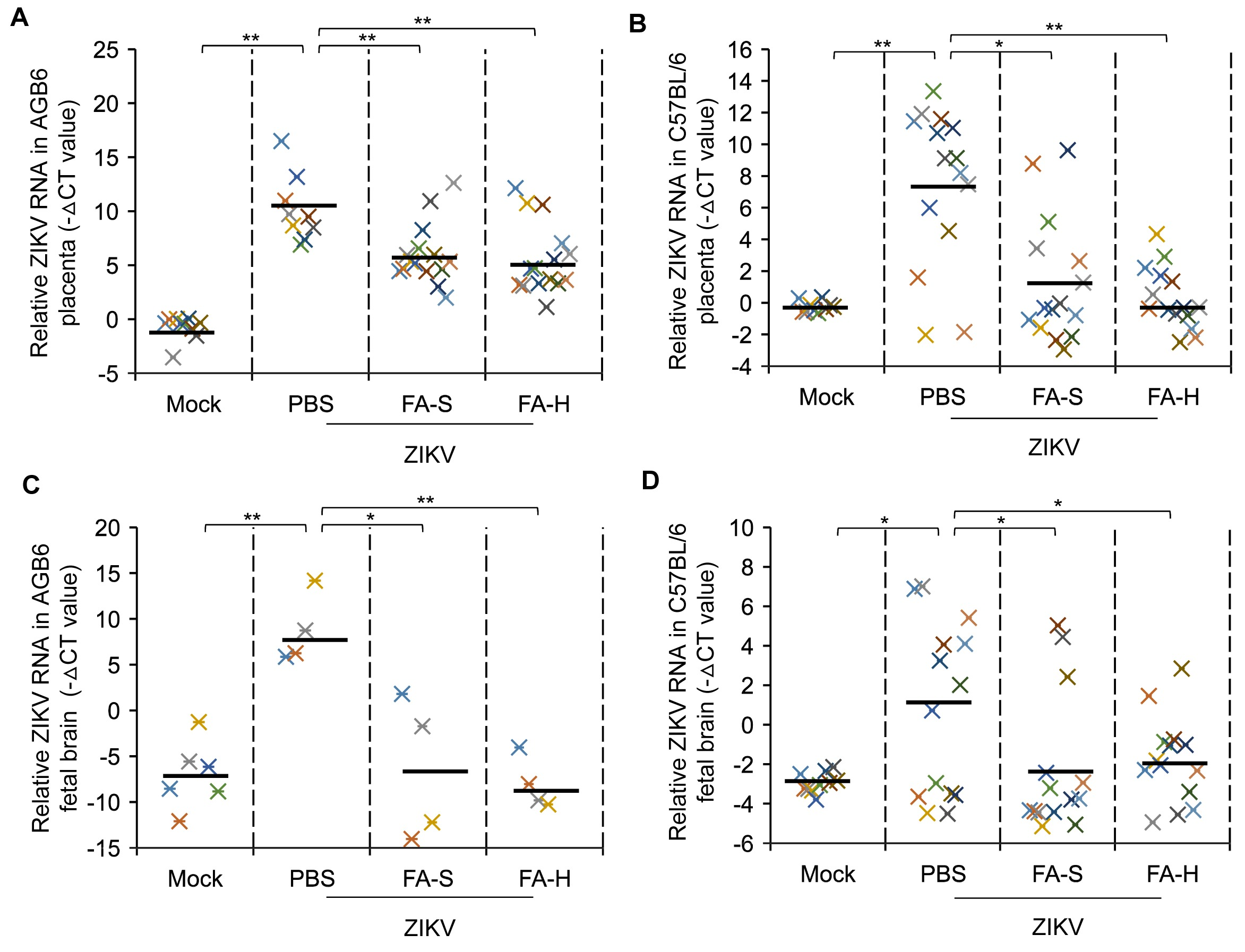

Supplement: S6 Fig — (A-B) Relative quantitative analysis of the ZIKV RNA levels in the individual placentae of AGB6 (A, E15.5) and C57BL/6 (B, E13.5) mice (n = 11–16 cDNA/group). (C-D) Relative quantitative analysis of ZIKV RNA levels in the fetal brain of AGB6 (C, E15.5) and C57BL/6 (D, E13.5) mice [n = pooled cDNA of 3–6 pregnant mice/ group (C) or 11–16 individual cDNA/group (D)]. Data are mean (black bar) and individual values. *P<0.05 and **P<0.01 by Kruskal-Wallis, Bonferroni post-hoc test. (TIF) [file ppat.1008521.s006.tif]

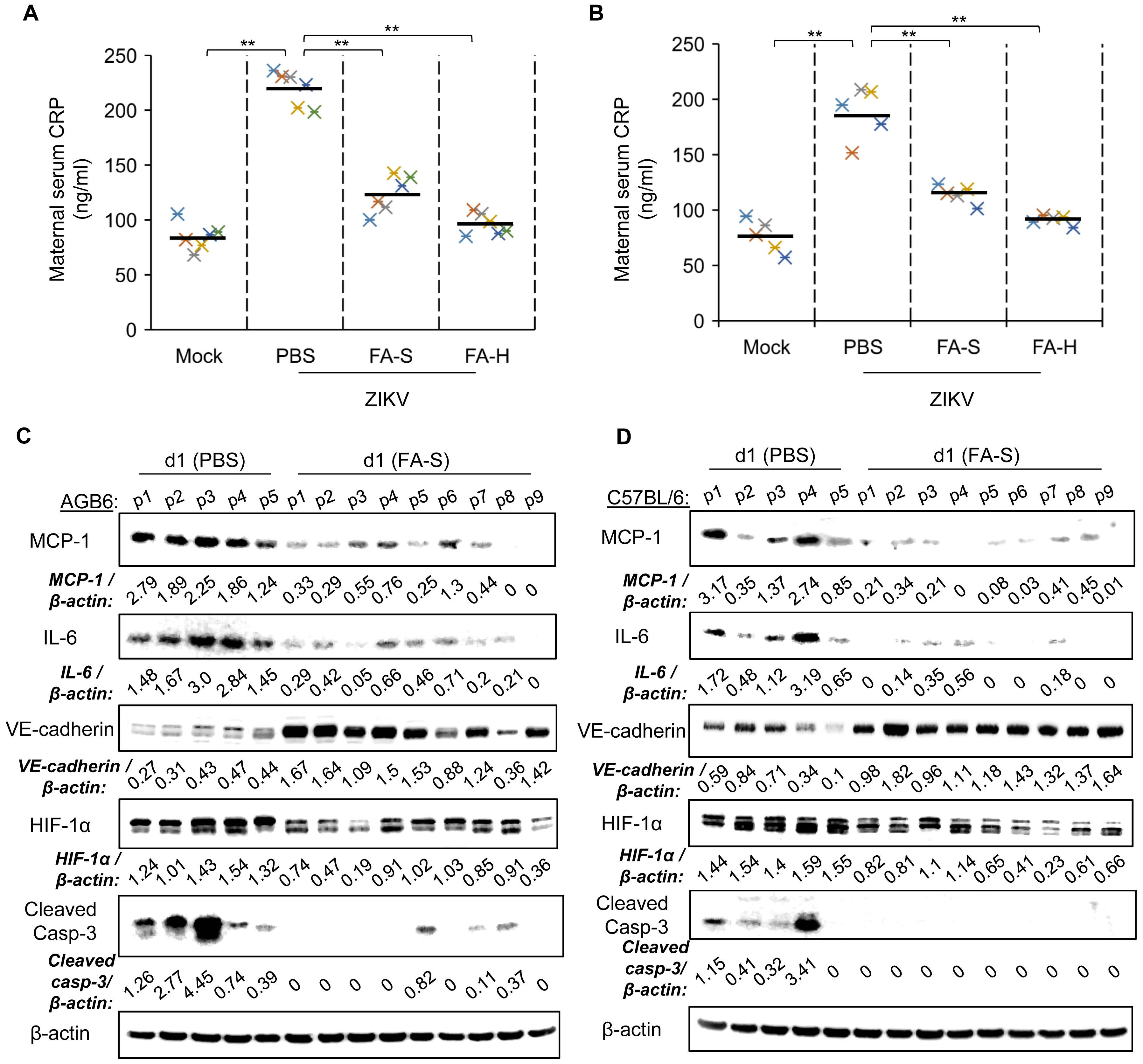

Supplement: S7 Fig — (A-B) The level of maternal serum CRP of ZIKV-infected pregnant AGB6 mice (A, E15.5) and ZIKV-infected pregnant C57BL/6 mice (B, E18.5). Data are mean (black bar) and individual values (n = 6 or 5 mice/group). **P<0.01 by Kruskal-Wallis, Bonferroni post-hoc test. (C-D) Western blot analysis of protein levels of inflammatory cytokines, VE-cadherin, HIF-1α, cleaved caspase-3 in the placenta of AGB6 (C) and C57BL/6 (D) mice, β-actin for loading control. Density ratios of respective protein and β-actin are shown in Western blot. Samples are individual placental tissue lysates (p1-p9) of 1 representative pregnant mouse (d1). (TIF) [file ppat.1008521.s007.tif]
