# Supplementary material for: Correction: Preventive effects of folic acid on Zika virus-associated poor pregnancy outcomes in immunocompromised mice
Source: PLoS Pathog. 2026 Jun 26;22(6):e1014363. doi: 10.1371/journal.ppat.1014363 (PMC13308848; doi:10.1371/journal.ppat.1014363)

Figure 7B (Correction: Mock)

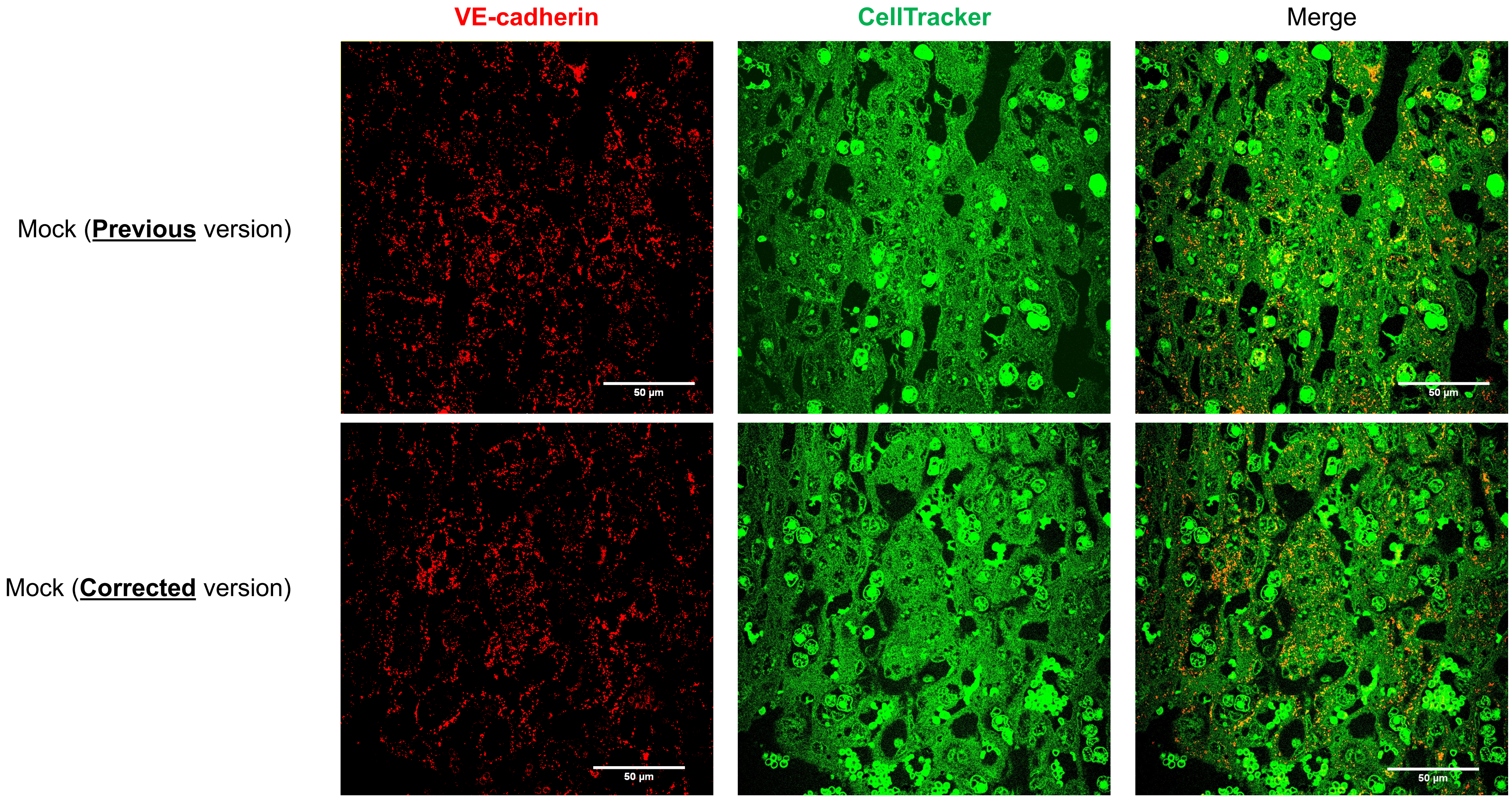

Figure 7B-Correction

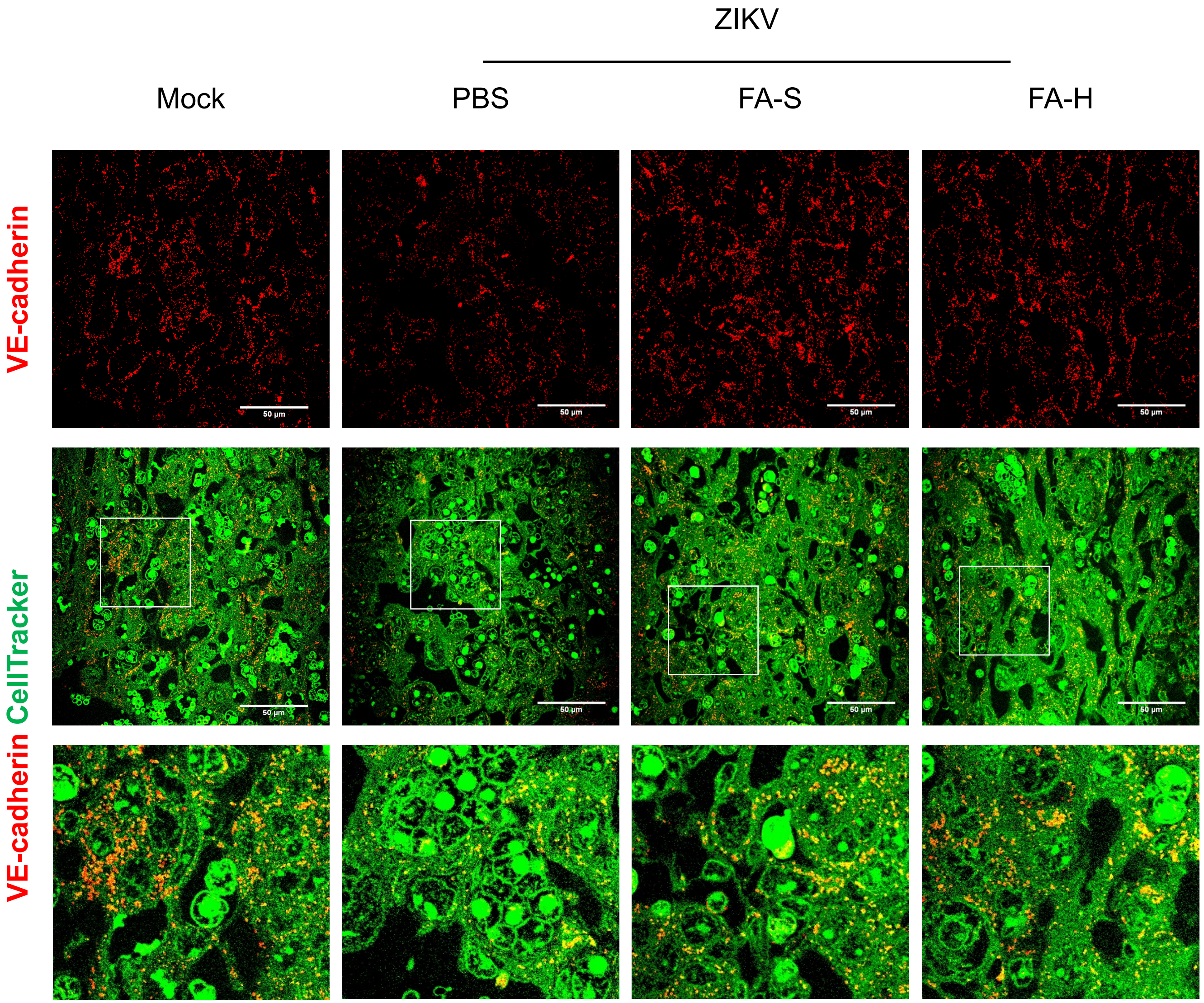

Supplement: S1 File — (ZIP) [file ppat.1014363.s001.zip › To sent/Fig 7B-Correction.pdf]
