# Supplementary figures and images for: Correction: Preventive effects of folic acid on Zika virus-associated poor pregnancy outcomes in immunocompromised mice
Source: PLoS Pathog. 2026 Jun 26;22(6):e1014363. doi: 10.1371/journal.ppat.1014363 (PMC13308848; doi:10.1371/journal.ppat.1014363)

# Figure 2C and 2D

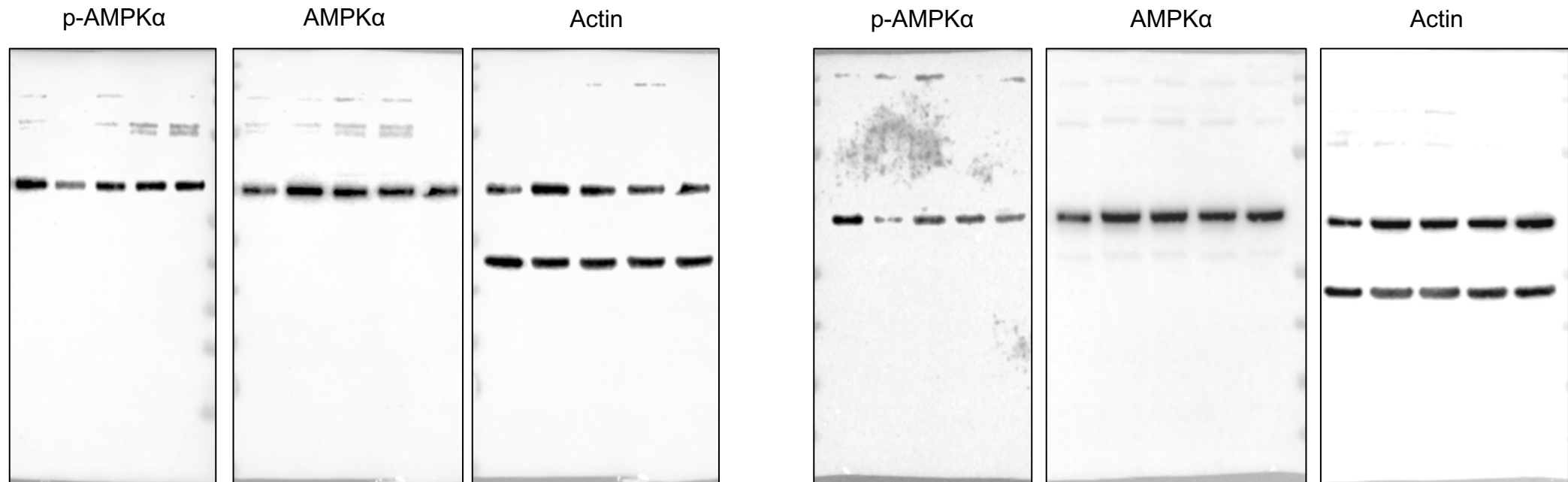

Supplement: S1 File — (ZIP) [file ppat.1014363.s001.zip › To sent/Fig 2C and 2D_Western blot.pdf]

# Figure 2F

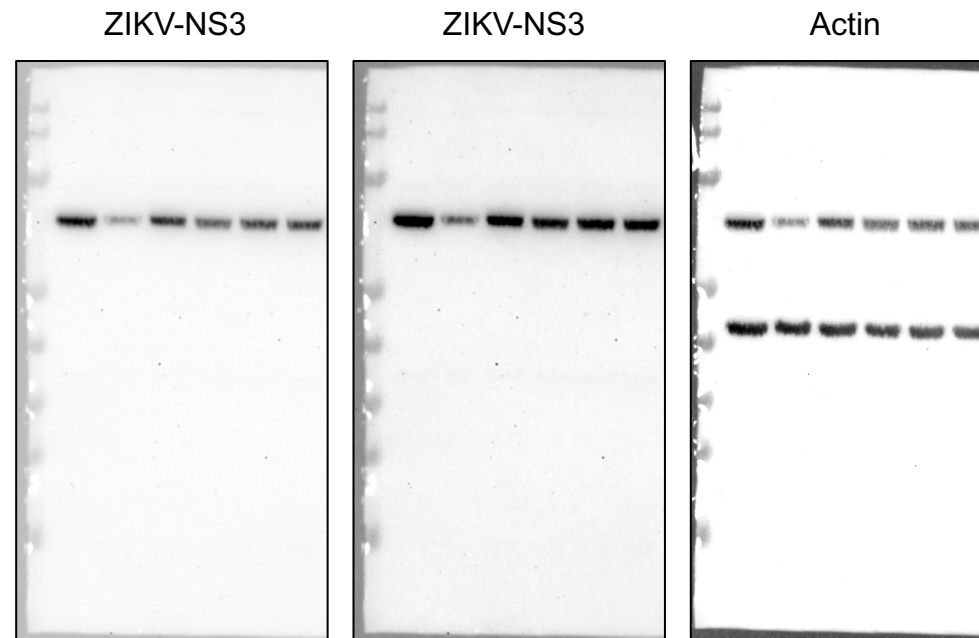

Supplement: S1 File — (ZIP) [file ppat.1014363.s001.zip › To sent/Fig 2F_Western blot.pdf]

# Figure 2E

ZIKV-NS3 Nuclei

shLacZ

shAMPK $\alpha$

shFR $\alpha$

DMSO

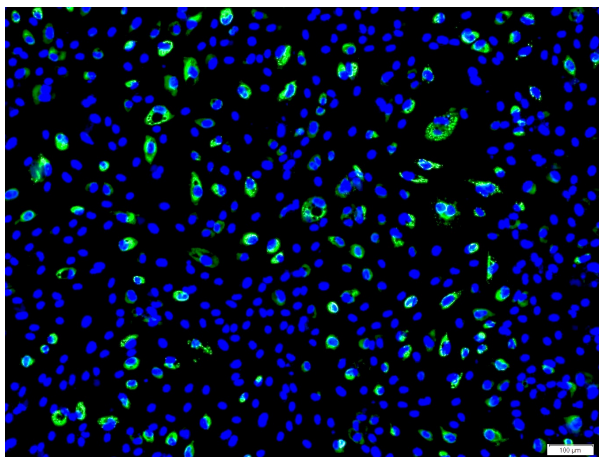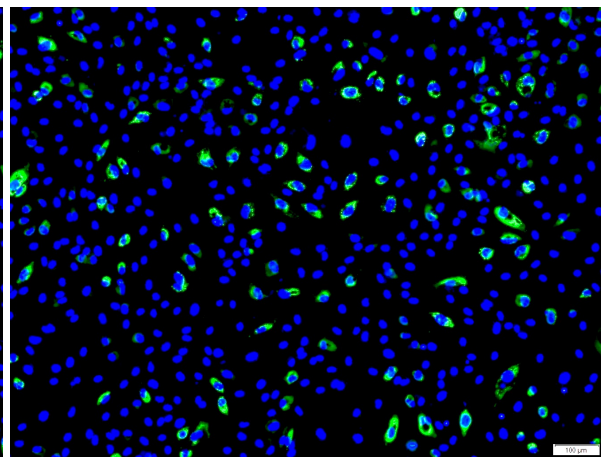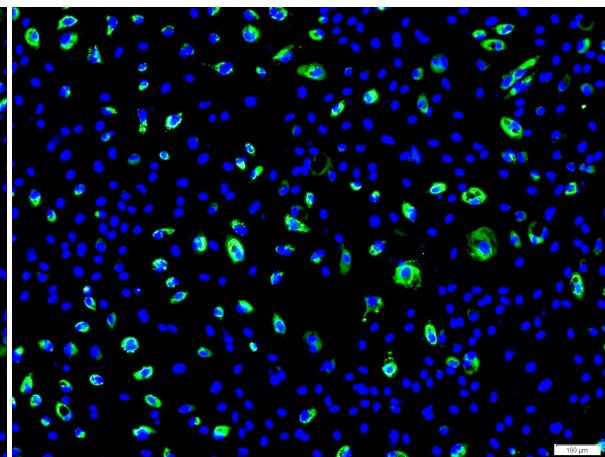

FA

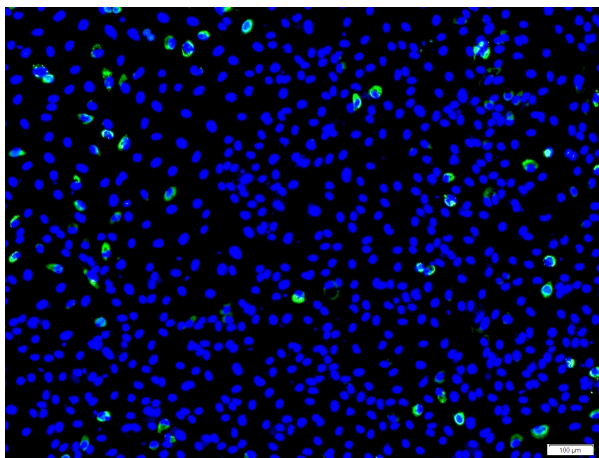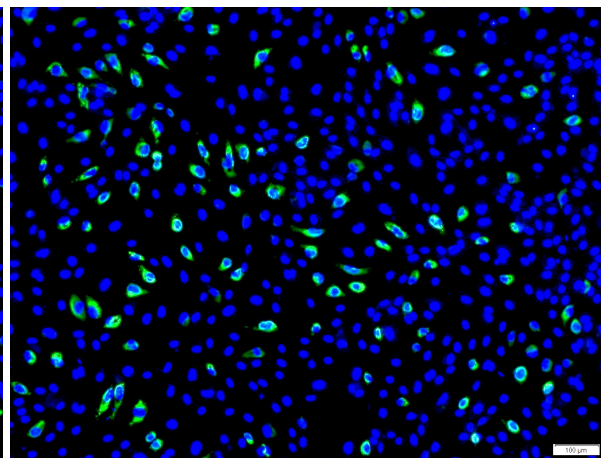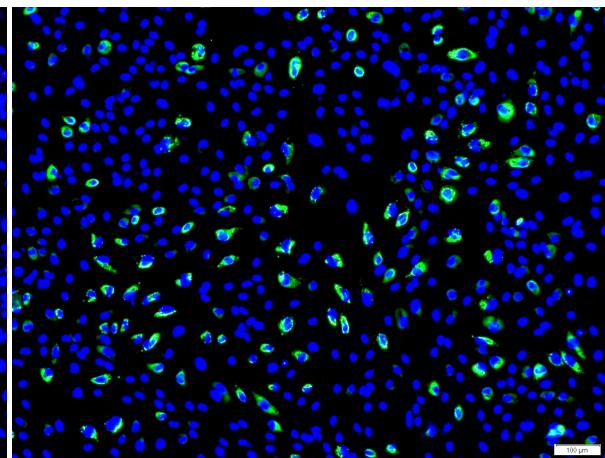

Supplement: S1 File — (ZIP) [file ppat.1014363.s001.zip › To sent/Fig 2E_Immunofluorescence image.pdf]

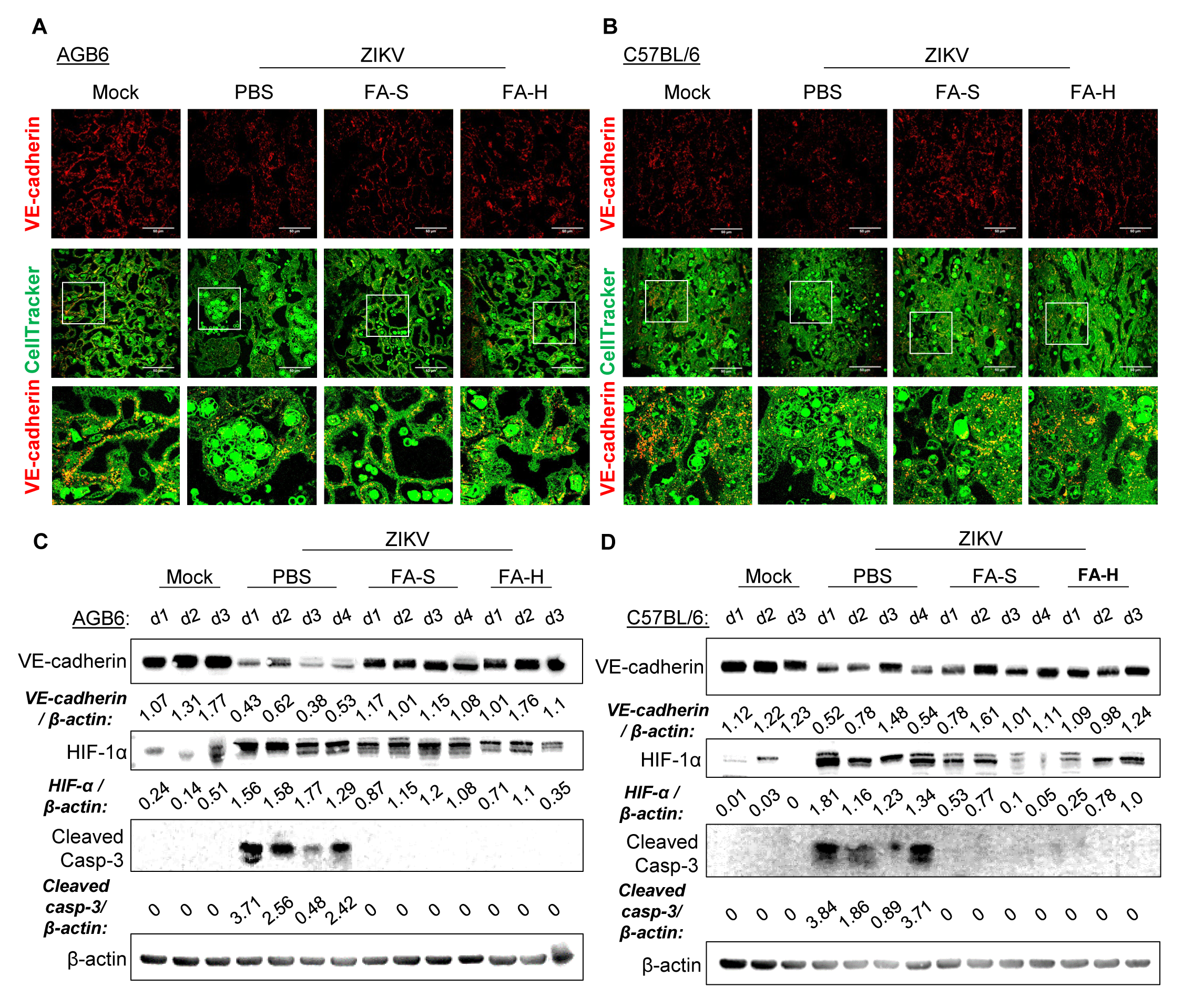

Supplement: S1 File — (ZIP) [file ppat.1014363.s001.zip › To sent/Fig 7-Corrected.tif]

# Figure S4H (Correction)

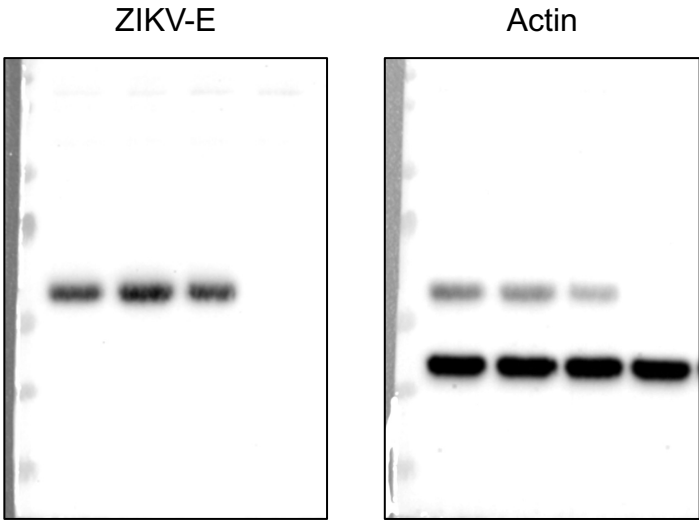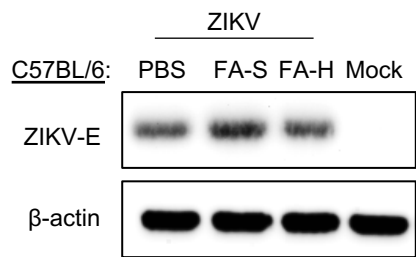

Supplement: S1 File — (ZIP) [file ppat.1014363.s001.zip › To sent/Fig S4H-Correction.pdf]

# Figure S4B

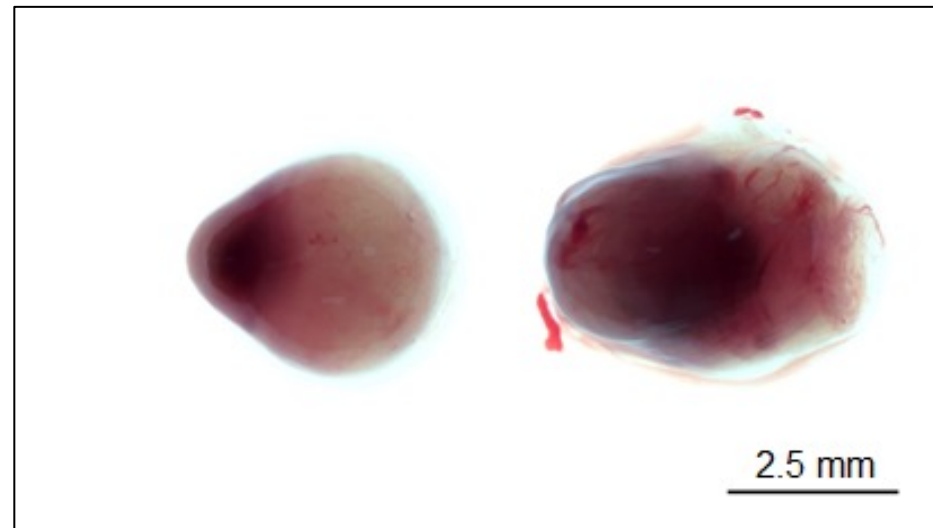

Supplement: S1 File — (ZIP) [file ppat.1014363.s001.zip › To sent/Fig S4B_Representative morphology of fetal resorption.pdf]

# Figure 7C

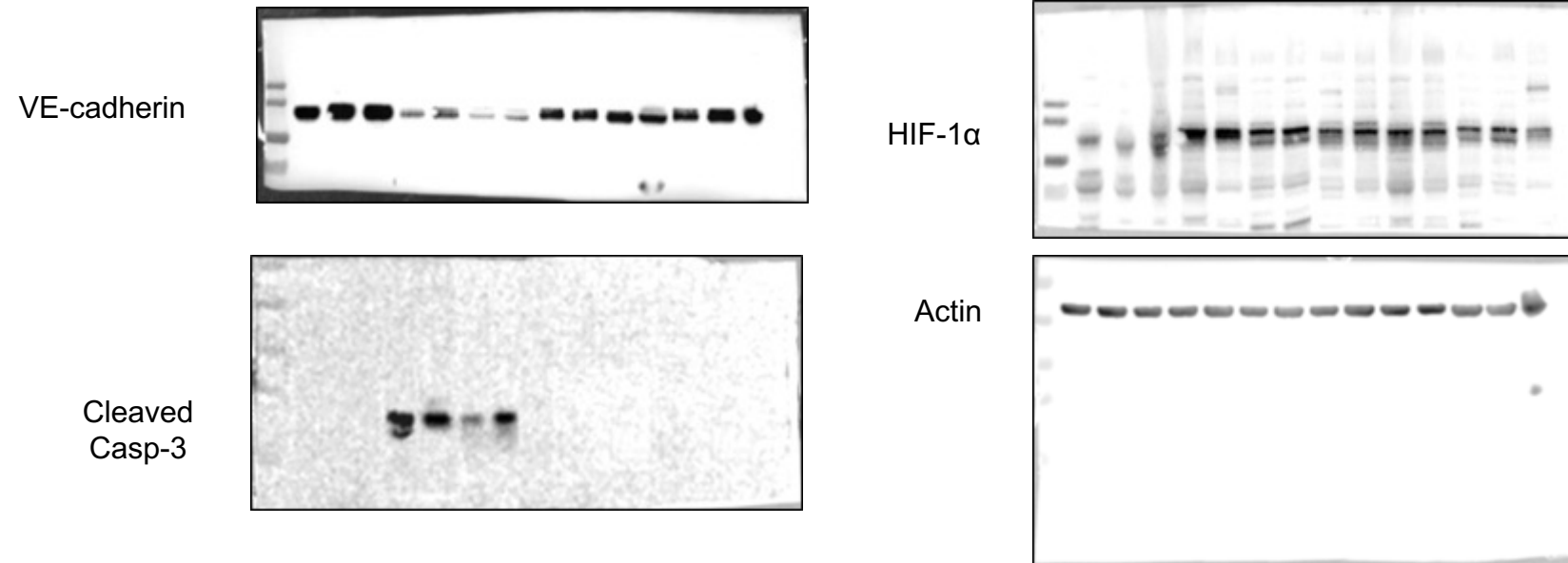

Supplement: S1 File — (ZIP) [file ppat.1014363.s001.zip › To sent/Fig 7C_Western blot.pdf]

# Figure S4C and S4D

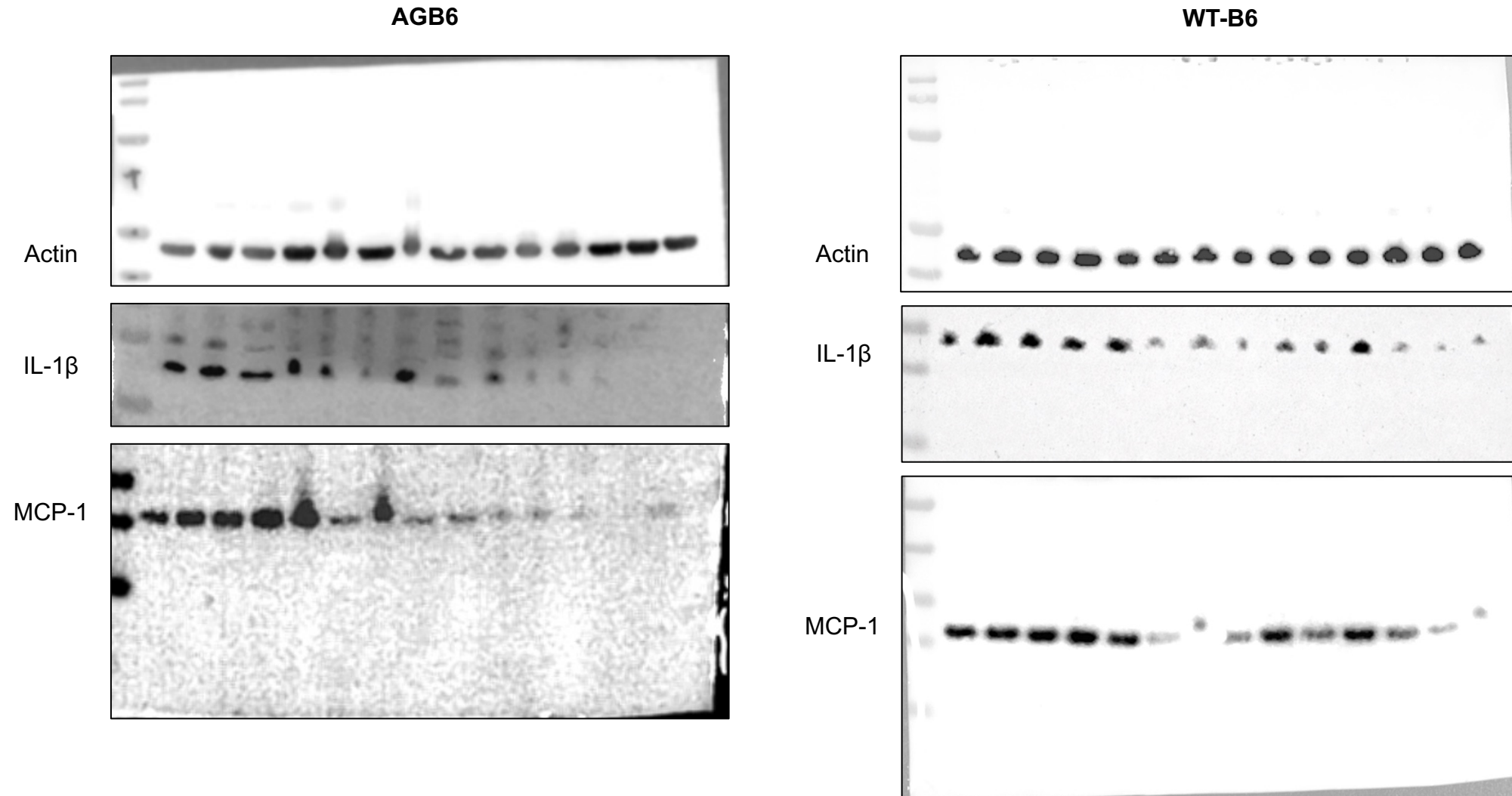

Supplement: S1 File — (ZIP) [file ppat.1014363.s001.zip › To sent/Fig S4C and S4D_Western blot.pdf]

# Figure 7D

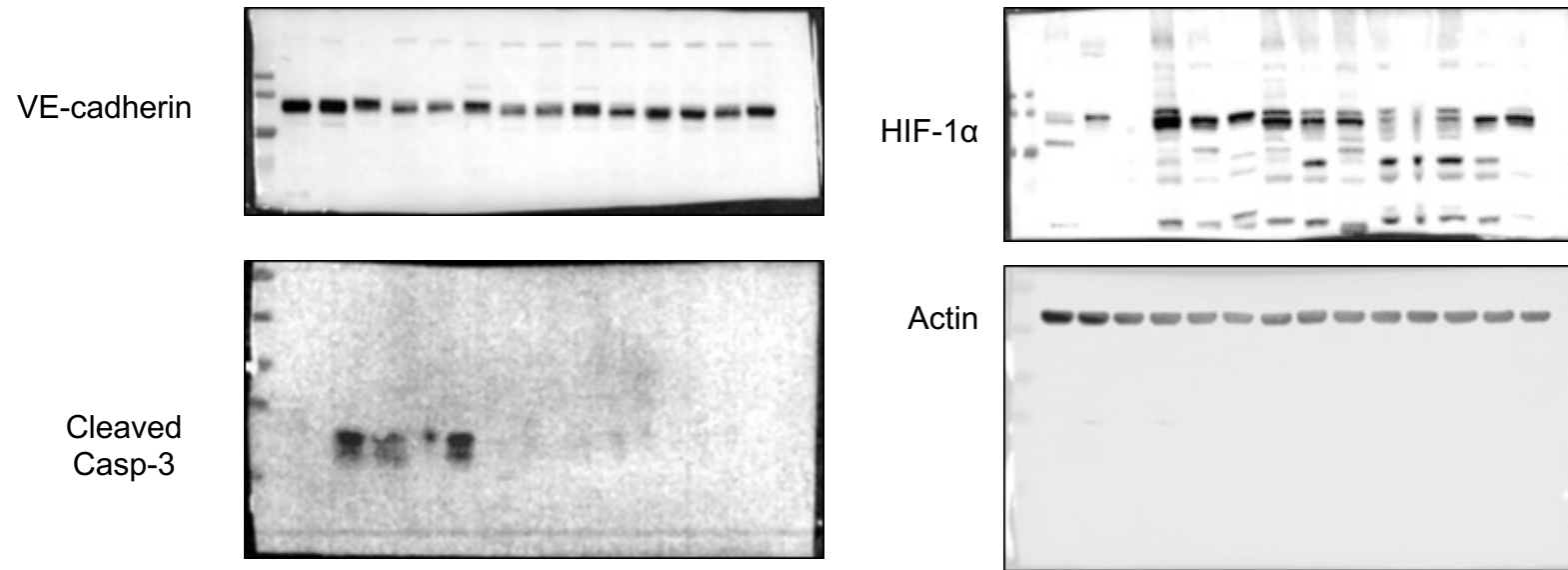

Supplement: S1 File — (ZIP) [file ppat.1014363.s001.zip › To sent/Fig 7D_Western blot.pdf]

# Figure S1E

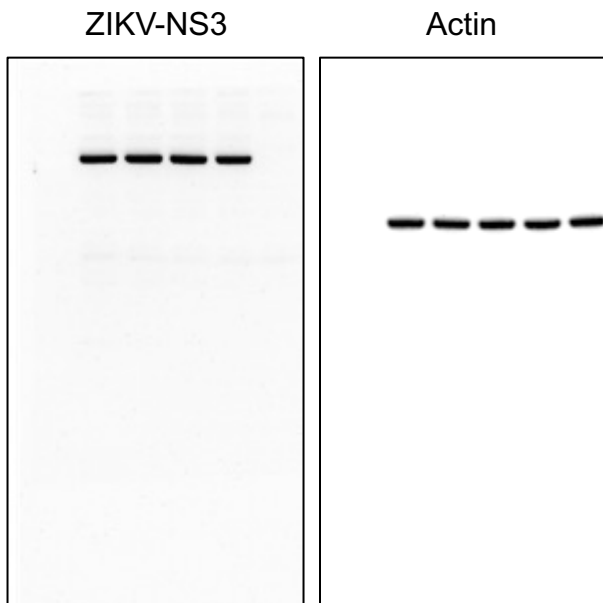

Supplement: S1 File — (ZIP) [file ppat.1014363.s001.zip › To sent/Fig S1E_Western blot.pdf]

# Figure S1B

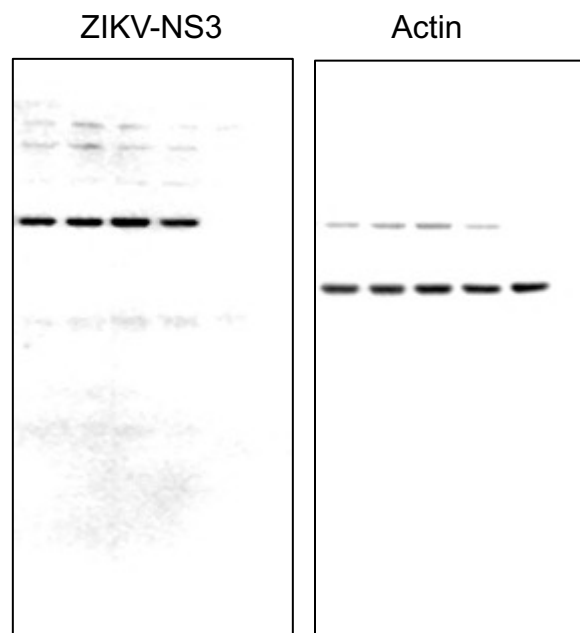

Supplement: S1 File — (ZIP) [file ppat.1014363.s001.zip › To sent/Fig S1B_Western blot.pdf]

# Figure S4H

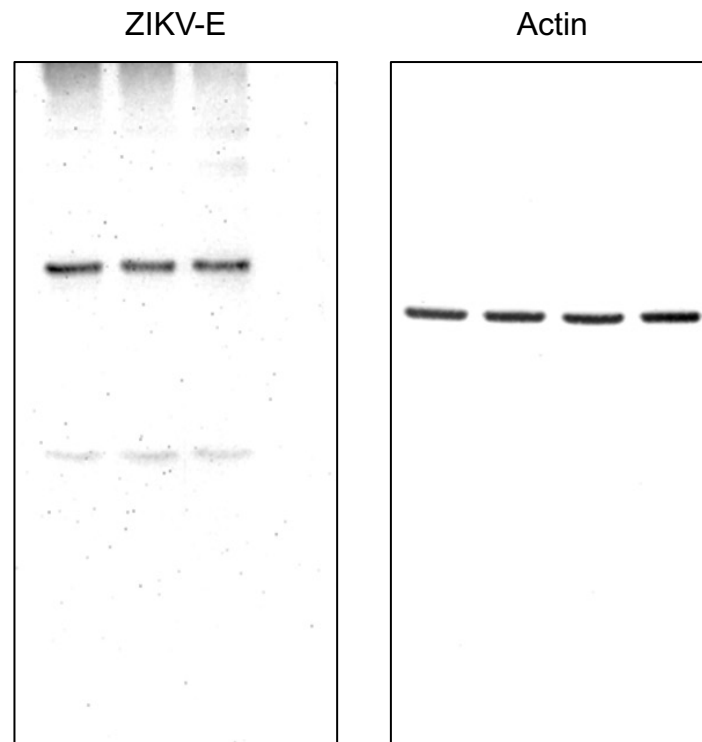

Supplement: S1 File — (ZIP) [file ppat.1014363.s001.zip › To sent/Fig S4H_Western blot.pdf]

# Figure S4F-G

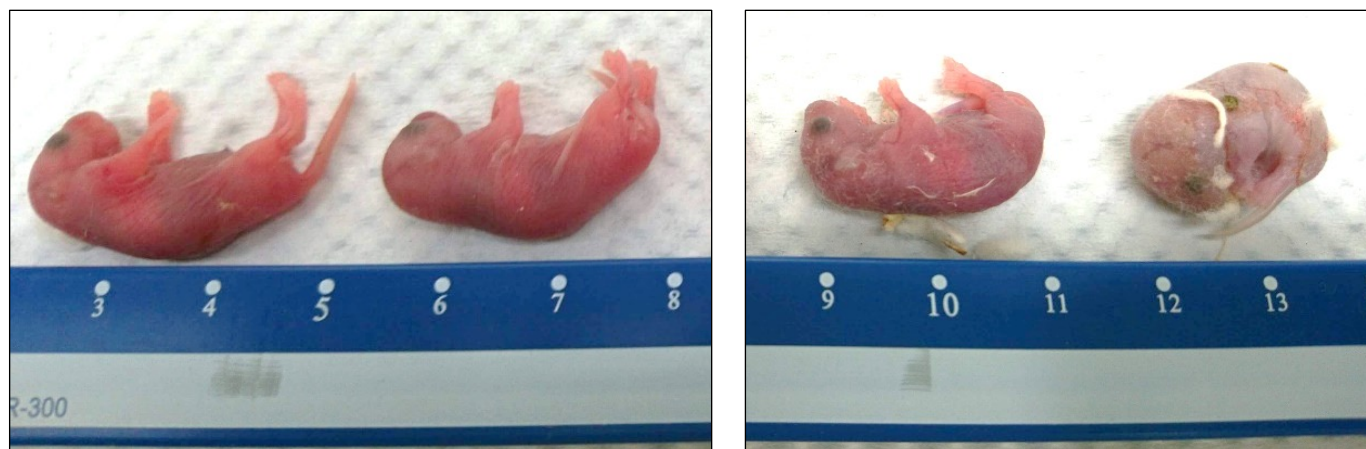

Supplement: S1 File — (ZIP) [file ppat.1014363.s001.zip › To sent/Fig S4F and S4G_Representative morphology of surviving pups.pdf]

# Figure 2B

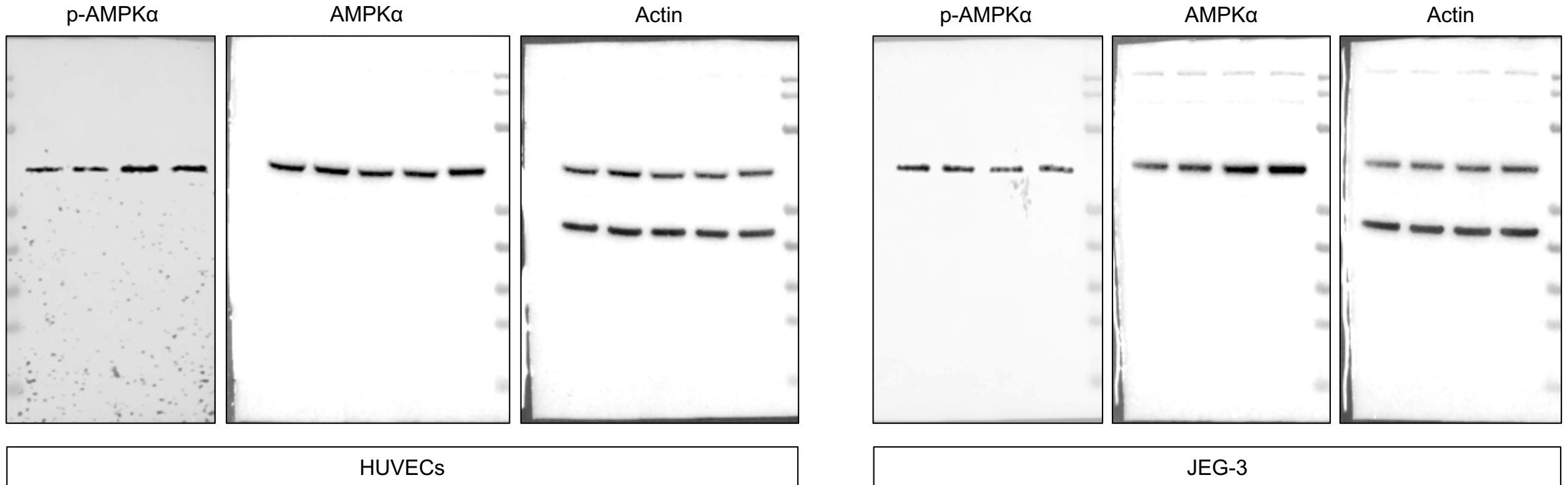

Supplement: S1 File — (ZIP) [file ppat.1014363.s001.zip › To sent/Fig 2B_Western blot.pdf]

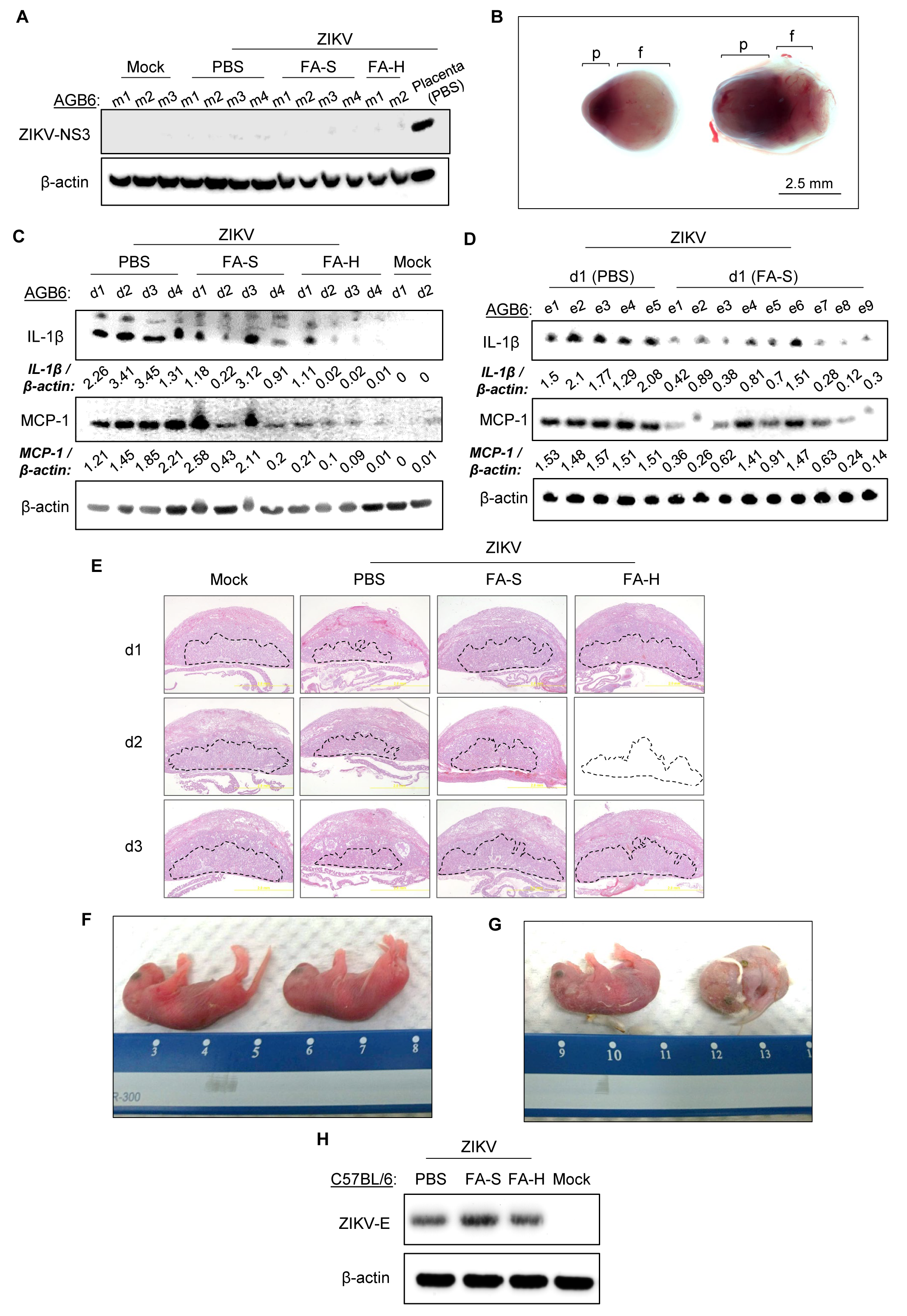

Supplement: S1 File — (ZIP) [file ppat.1014363.s001.zip › To sent/Fig S4-Corrected.tif]

# Figure S4A

ZIKV-NS3

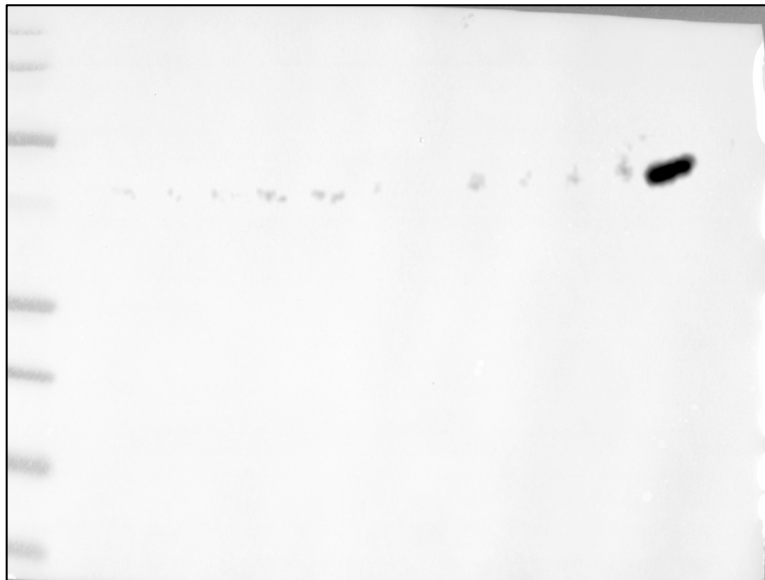

Actin

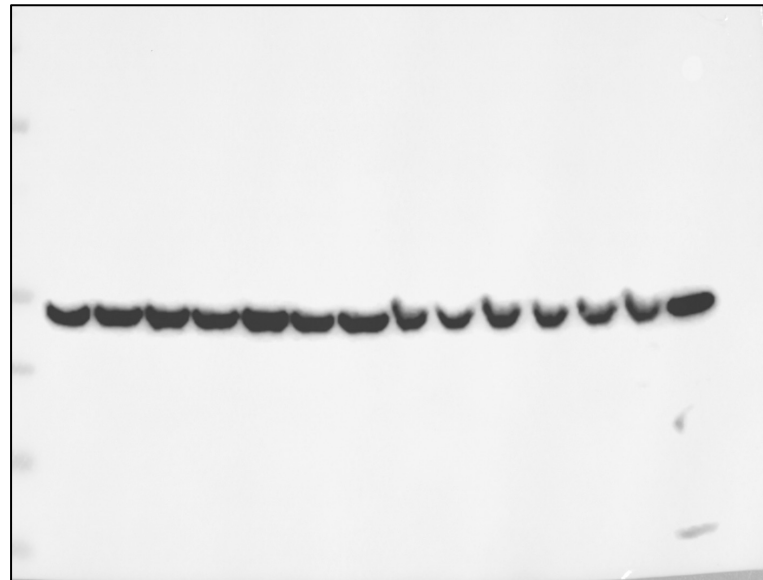

Supplement: S1 File — (ZIP) [file ppat.1014363.s001.zip › To sent/Fig S4A_Western blot.pdf]

# Figure 5C

Mock

ZIKV+PBS

ZIKV+FA-S

ZIKV+FA-H

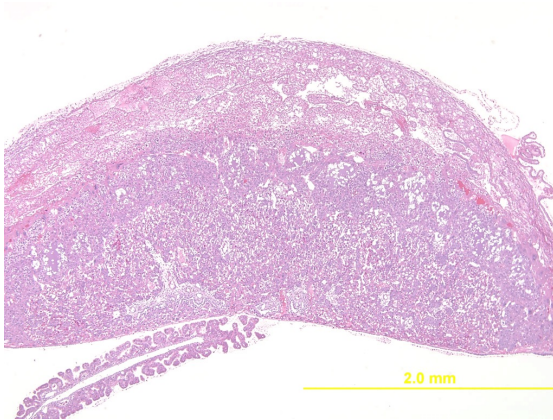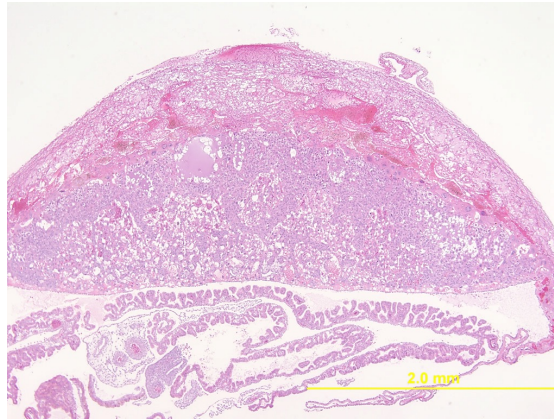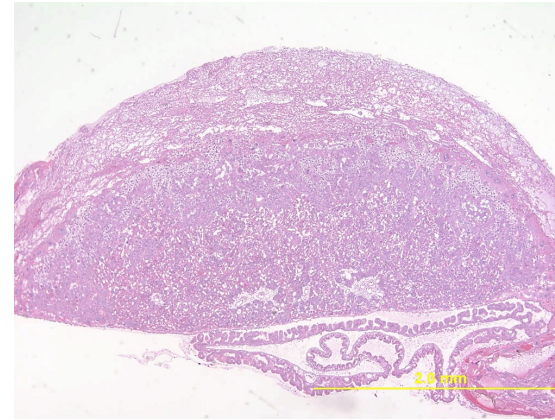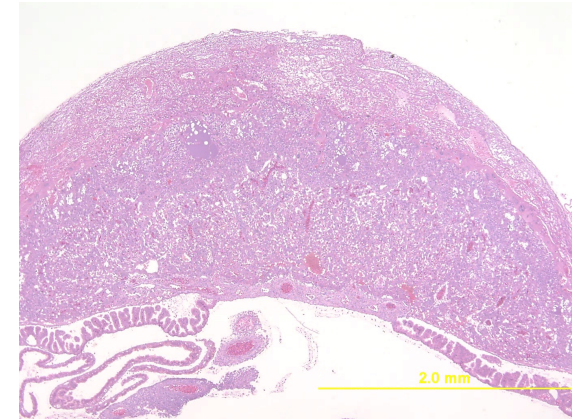

Supplement: S1 File — (ZIP) [file ppat.1014363.s001.zip › To sent/Fig 5C_Histological image of placentae.pdf]

# Figure S1D

ZIKV-NS3 Nuclei

ZIKV

FA ( $\mu\text{M}$ )

Mock

DMSO

1.56

3.125

6.25

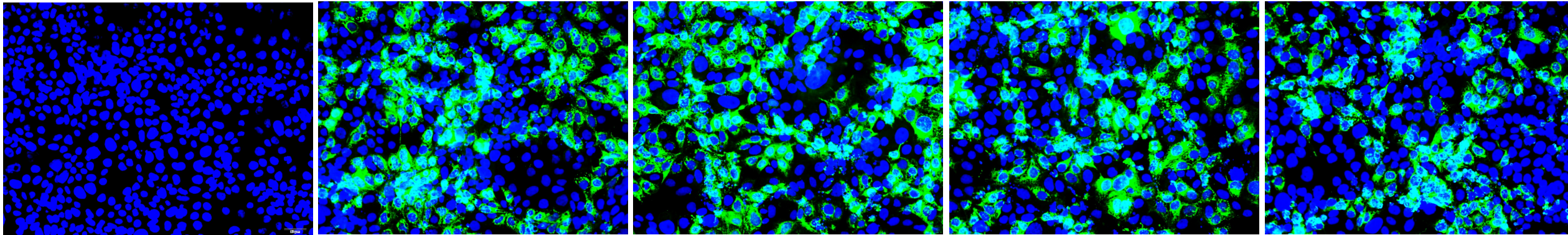

Supplement: S1 File — (ZIP) [file ppat.1014363.s001.zip › To sent/Fig S1D_Immunofluorescence image.pdf]

# Figure S1A

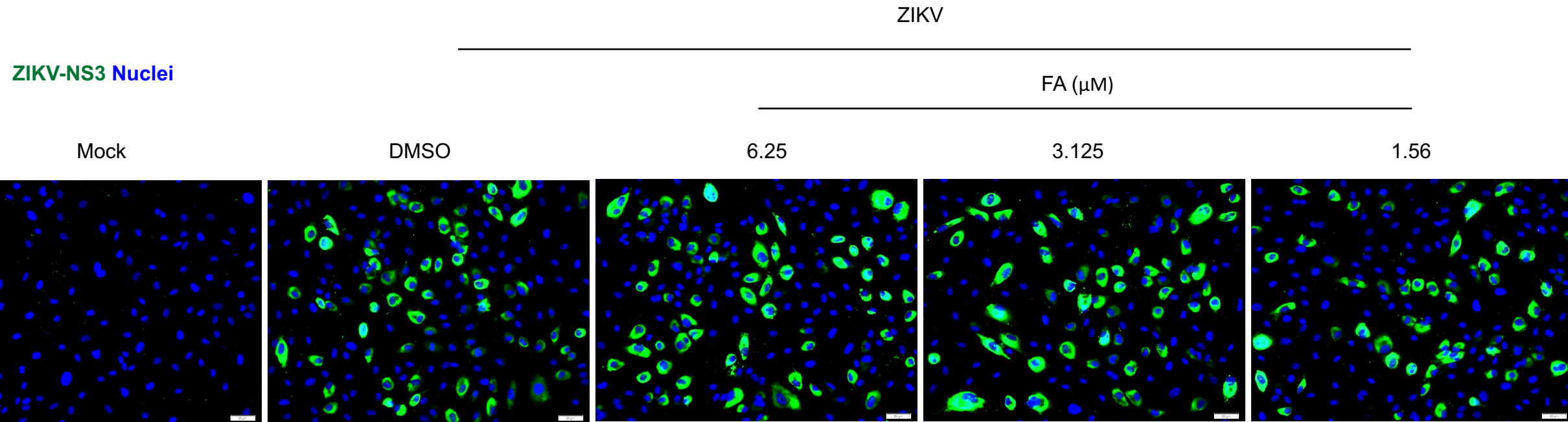

# Figure S1B

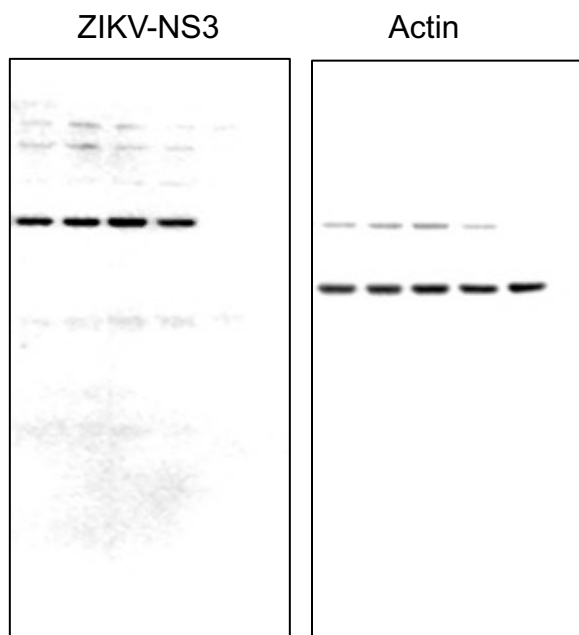

# Figure S1D

ZIKV-NS3 Nuclei

ZIKV

FA ( $\mu\text{M}$ )

Mock

DMSO

1.56

3.125

6.25

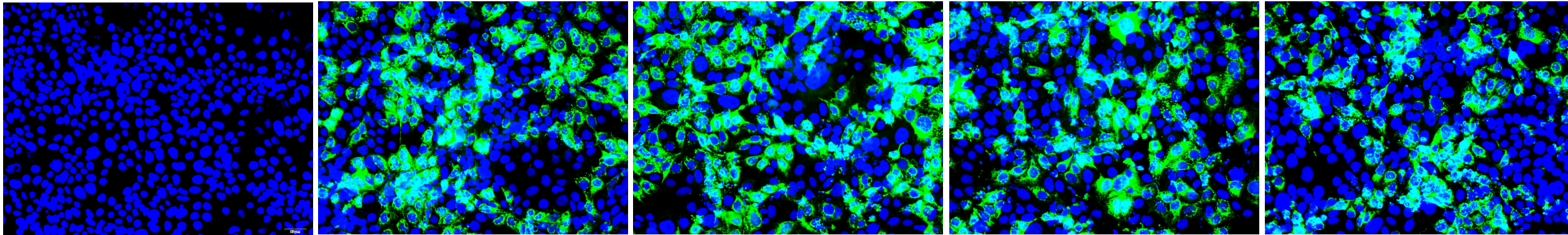

# Figure S1E

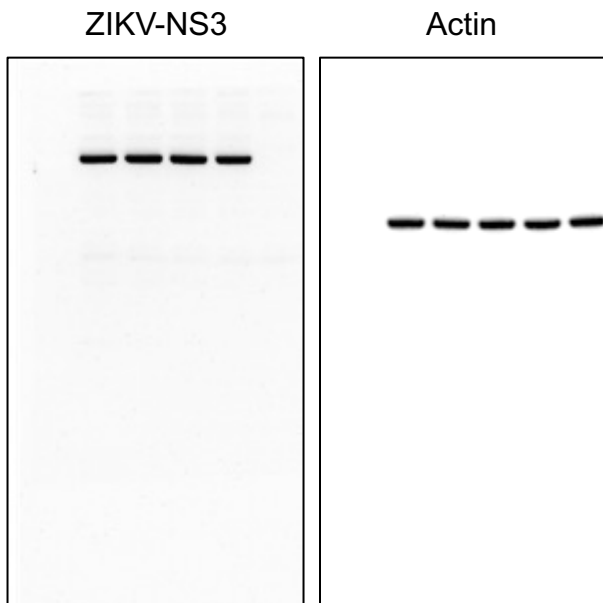

Supplement: S1 File — (ZIP) [file ppat.1014363.s001.zip › To sent/Fig S1A_Immunofluorescence image.pdf]

# Figure S4E

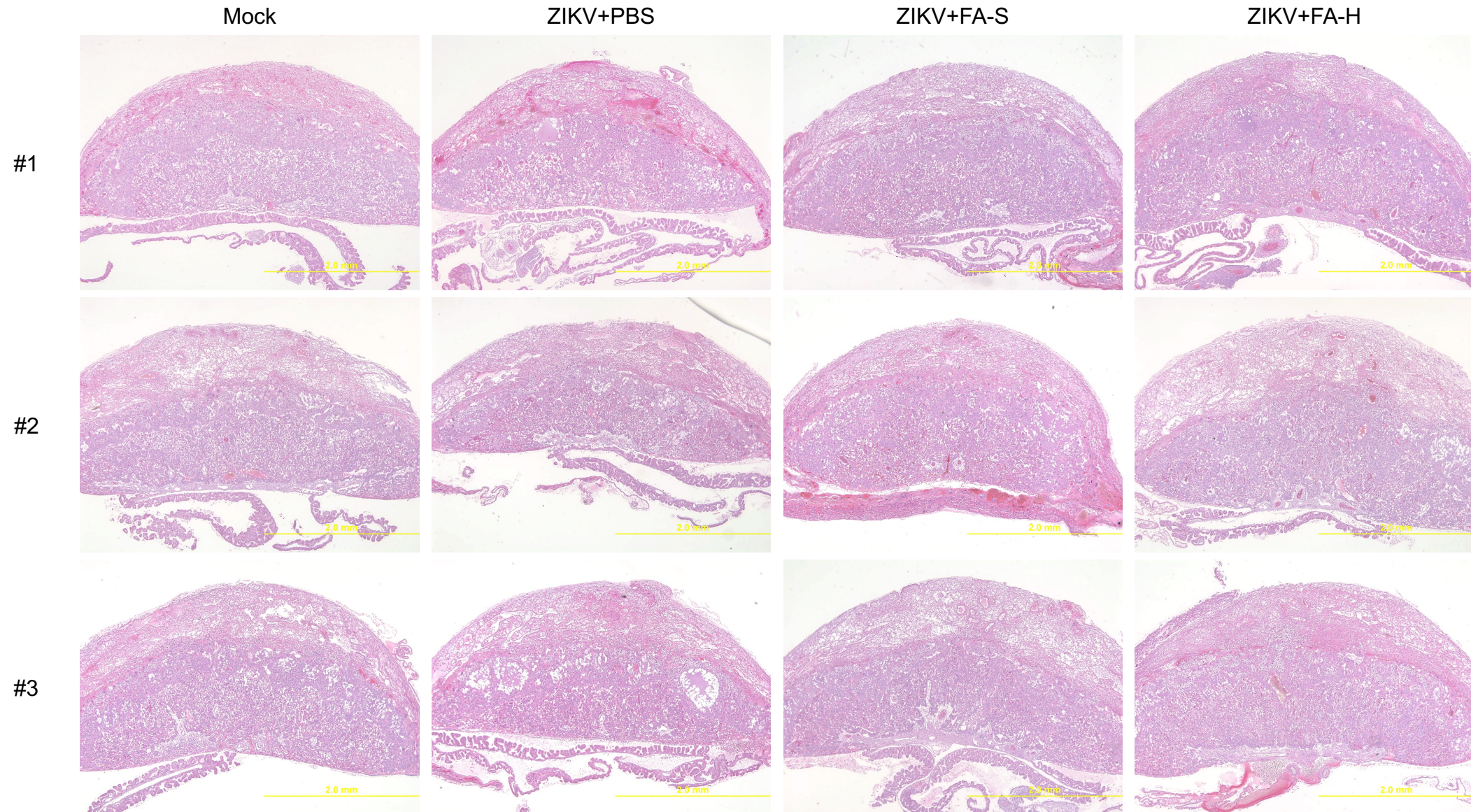

Supplement: S1 File — (ZIP) [file ppat.1014363.s001.zip › To sent/Fig S4E_Histological image of placentae.pdf]

# Figure 2A

HUVECs

JEG-3

FR $\alpha$  Nuclei

FR $\alpha$  Nuclei

FOLT Nuclei

FOLT Nuclei

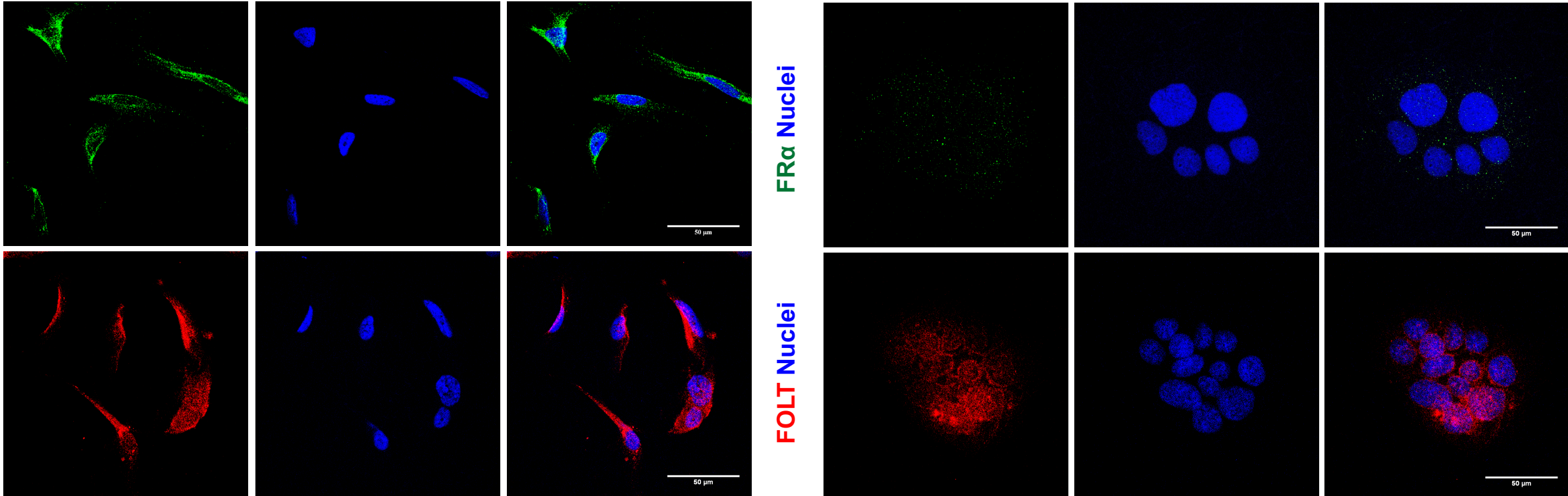

Supplement: S1 File — (ZIP) [file ppat.1014363.s001.zip › To sent/Fig 2A_Confocal image.pdf]

# Figure 7A

AGB6

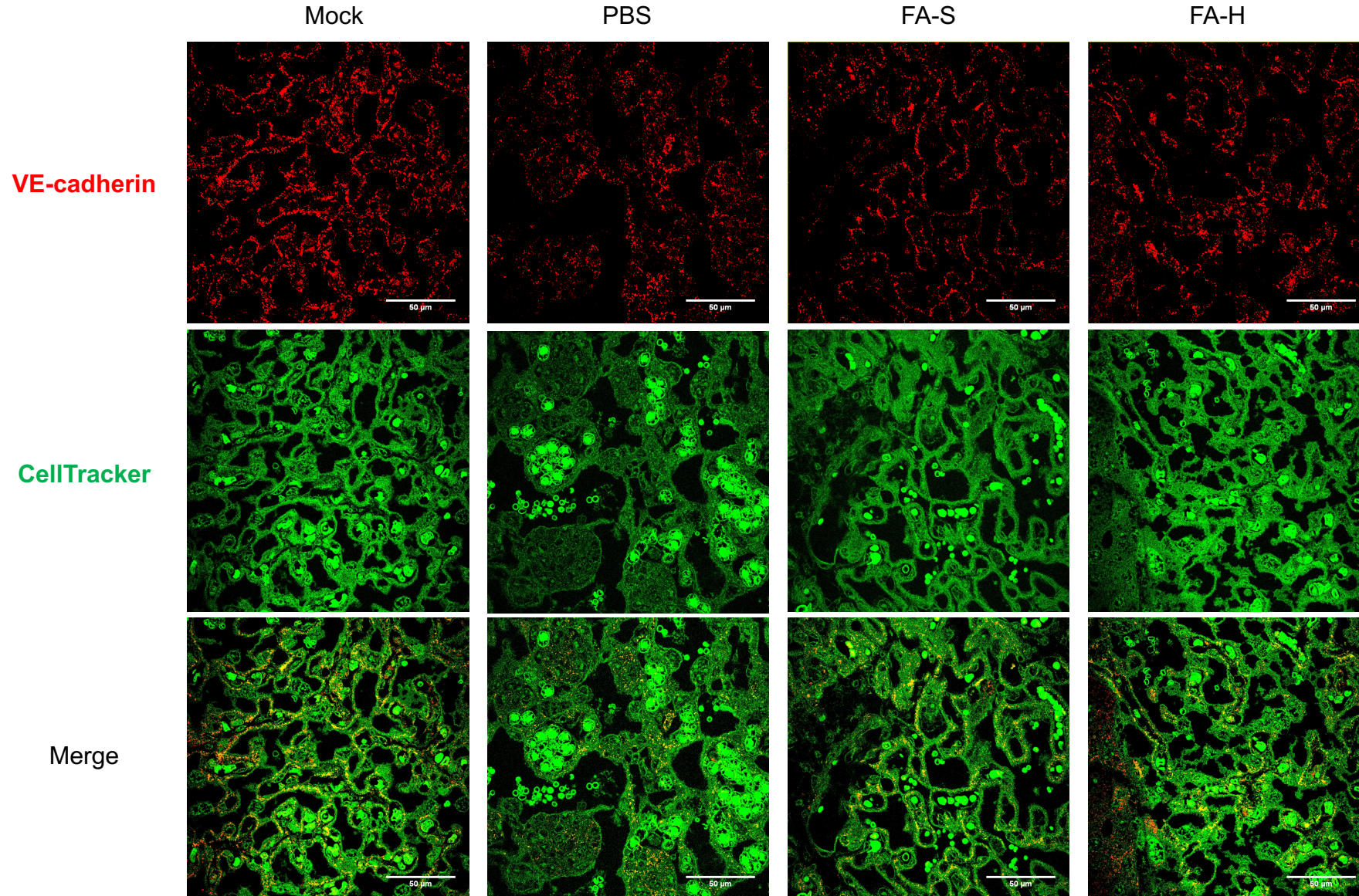

Supplement: S1 File — (ZIP) [file ppat.1014363.s001.zip › To sent/Fig 7A_Immunofluorescence image.pdf]

# Figure 7B

C57BL/6

Mock

PBS

FA-S

FA-H

VE-cadherin

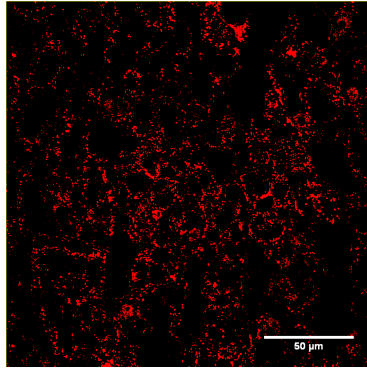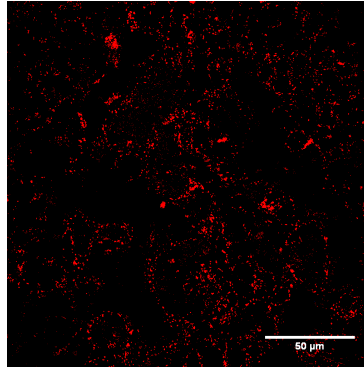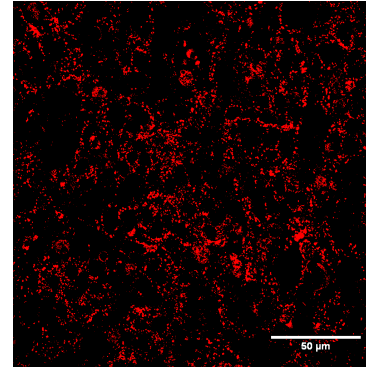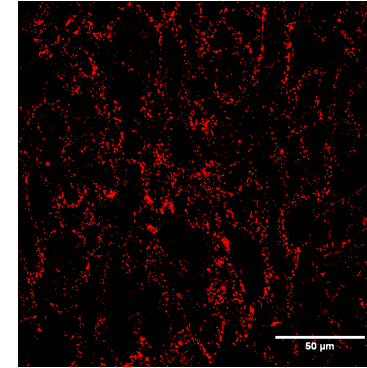

CellTracker

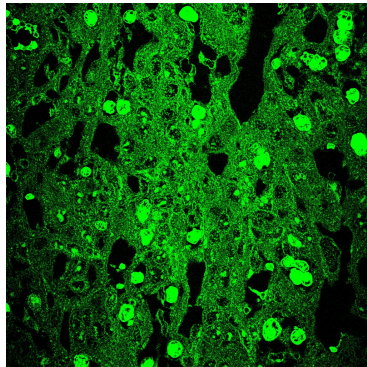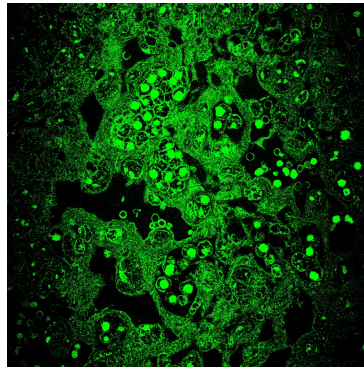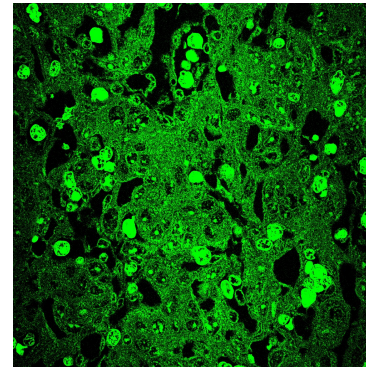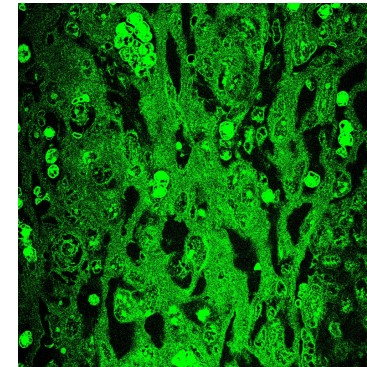

Merge

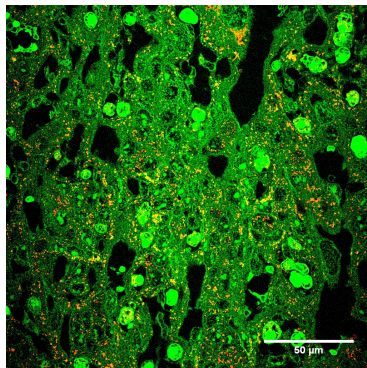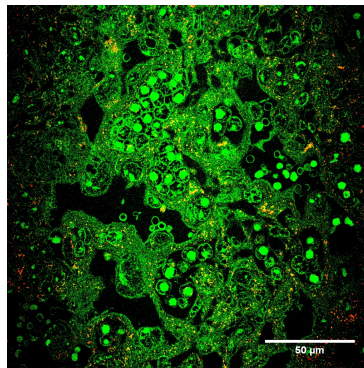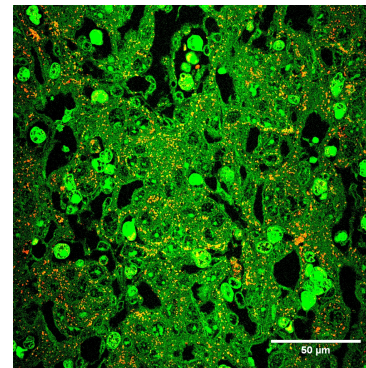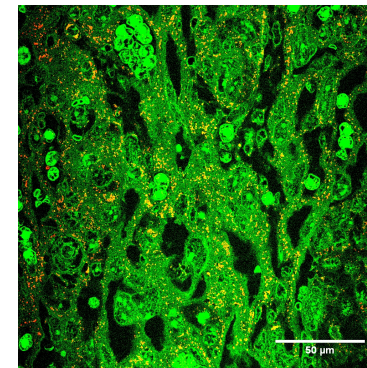

Supplement: S1 File — (ZIP) [file ppat.1014363.s001.zip › To sent/Fig 7B_Immunofluorescence image.pdf]
